# Prednisolone and Ketorolac vs Ketorolac Monotherapy or Sub-Tenon Prophylaxis for Macular Thickening in Cataract Surgery: A Randomized Clinical Trial

**DOI:** 10.1001/jamaophthalmol.2021.2976

**Published:** 2021-08-12

**Authors:** Jesper Høiberg Erichsen, Lars M. Holm, Mads Forslund Jacobsen, Julie L. Forman, Line Kessel

**Affiliations:** 1 Department of Ophthalmology, Rigshospitalet Glostrup, Glostrup, Denmark; 2 Department of Clinical Medicine, University of Copenhagen, Copenhagen, Denmark; 3 Section of Biostatistics, Department of Public Health, University of Copenhagen, Copenhagen, Denmark

## Abstract

**Importance:**

The choice of anti-inflammatory prophylaxis parallel to cataract surgery is important for patient safety and successful outcome of surgery, but which regimen to choose is contested.

**Objectives:**

To determine whether a combination of prednisolone and nonsteroidal anti-inflammatory drug (NSAID) eye drops was superior in preventing increased central macular thickness (central subfield thickness [CST]) after uncomplicated cataract surgery compared with NSAID monotherapy and sub-Tenon capsule depot (dropless surgery), and to test whether preoperative initiation of eye drop treatment was superior to initiation on the day of surgery.

**Design, Setting, and Participants:**

This investigator-driven, single-center, randomized clinical trial with masked statistical analyses enrolled patients at the Department of Ophthalmology, Rigshospitalet Glostrup, Glostrup, Denmark, from February 1, 2018, to August 15, 2019. Follow-up was completed December 18, 2019. Participants included low-risk patients undergoing phacoemulsification for age-related cataract by an experienced surgeon (1 eye per participant). Data were analyzed from February 17 to June 15, 2020.

**Interventions:**

Participants scheduled for cataract removal were randomized to 1 of 5 anti-inflammatory prophylactic regimens: eye drops with a combination of prednisolone, 1%, and ketorolac tromethamine, 0.5%, with or without preoperative initiation (preoperative prednisolone plus NSAID [control] and postoperative prednisolone plus NSAID groups), ketorolac monotherapy with or without preoperative initiation (preoperative and postoperative NSAID groups), or sub-Tenon depot of dexamethasone phosphate (sub-Tenon group). Eye drops were administered 3 times per day until 3 weeks postoperatively.

**Main Outcomes and Measures:**

CST 3 months postoperatively.

**Results:**

A total of 470 participants (mean [SD] age, 72.2 [7.0] years; 290 women [61.7%]) with 94 participants in each group were included in the analysis. Three months after surgery, the mean CST was 250.7 (95% CI, 247.6-253.7) μm in the preoperative prednisolone plus NSAID group, 250.7 (95% CI, 247.8-253.7) μm in the postoperative prednisolone plus NSAID group, 251.3 (95% CI, 248.2-254.4) μm in the preoperative NSAID group, 249.2 (95% CI, 246.2-252.3) μm in the postoperative NSAID group, and 255.2 (95% CI, 252.0-258.3) μm in the sub-Tenon group. There were no significant differences in CST or visual acuity compared with control and no differences between preoperative and postoperative groups, but 47 of 83 participants (56.6%) in the sub-Tenon group needed additional anti-inflammatory treatment.

**Conclusions and Relevance:**

No differences in CST or visual acuity were detected between the combination of prednisolone and NSAID eye drops vs NSAID monotherapy or sub-Tenon dexamethasone depot, although more than one-half of patients in the sub-Tenon arm received additional anti-inflammatory treatment. Initiating prophylaxis 3 days preoperatively was not superior to initiation on the day of surgery. Monotherapy with NSAIDs may be preferred in uncomplicated cataract surgery.

**Trial Registration:**

ClinicalTrials.gov Identifier: NCT03383328

## Key Points

**Question:**

Is a combination of corticosteroid and nonsteroidal anti-inflammatory drug (NSAID) eye drops superior to NSAID eye drops alone or dropless surgery with a sub-Tenon dexamethasone depot in controlling postoperative central macular thickening after uncomplicated cataract surgery?

**Findings:**

In this randomized clinical trial with 470 participants, no differences in central subfield thickness or visual acuity across treatment arms were identified, although approximately half of the group given the sub-Tenon depot received additional anti-inflammatory treatment.

**Meaning:**

Therapy with NSAID plus corticosteroid eye drops was not superior to NSAID monotherapy or sub-Tenon depot for postoperative central macular thickening after uncomplicated cataract surgery.

## Teaser

This randomized clinical trial assesses whether corticosteroid plus nonsteroidal anti-inflammatory drug (NSAID) eye drops were superior in preventing macular thickening compared with NSAID monotherapy and/or a sub-Tenon dexamethasone depot among patients undergoing uncomplicated cataract surgery.

## Introduction

Pseudophakic cystoid macular edema (PCME) is an important cause of unsatisfactory visual outcome after cataract surgery. It affects visual acuity after approximately 1% of surgical procedures.^1,2^ There is no common classification of PCME, and therefore estimates differ between studies. A meta-analysis^3^ reported that 4% to 25% had some degree of macular edema, even when prophylactic treatment was administered. It is believed that PCME is caused by the inflammatory response after cataract surgery, which disrupts the blood-ocular barrier leading to leakage of fluid into the retina.^4^ To control the inflammatory response and reduce the risk of PCME, prophylactic anti-inflammatory eye drops are prescribed parallel to cataract surgery.

The choice of anti-inflammatory prophylaxis is contested.^5,6^ Two types of drugs are available: corticosteroids and nonsteroidal anti-inflammatory drugs (NSAIDs). Corticosteroids inhibit phospholipases and thereby both prostaglandin and lipoxin synthesis,^7^ and NSAIDs selectively inhibit cyclooxygenase enzymes, which convert arachidonic acids to prostaglandins^7^; however, meta-analyses^3,5,6,8^ have found that NSAIDs are more effective in preventing PCME. It has been suggested that preoperative initiation of NSAID prophylaxis is beneficial,^9,10,11^ but to our knowledge, evidence from large randomized trials is missing.

Prophylactic treatment parallel to cataract surgery is traditionally administered as eye drops. Many patients have difficulties using eye drops and need assistance.^12^ Therefore, several dropless approaches have been suggested, and some studies^2,13,14^ have found that the effectiveness of these dropless approaches is comparable to corticosteroid eye drop monotherapy. To our knowledge, no studies have compared a dropless approach with NSAID eye drops. The purpose of this study was to determine whether (1) combination of prednisolone and NSAID eye drops is superior in preventing macular thickening after uncomplicated cataract surgery compared with NSAID monotherapy and dropless surgery using a sub–Tenon capsule dexamethasone phosphate depot and (2) preoperative initiation of eye drop treatment is superior to initiating treatment on the day of surgery.

## Methods

### Design and Intervention

This study was a randomized clinical trial comparing 5 different regimens for anti-inflammatory prophylaxis parallel to uncomplicated cataract surgery. The trial protocol and statistical analysis plan are found in Supplement 1 and Supplement 2, respectively. The control group received a combination of corticosteroid and NSAID eye drops, initiated 3 days before surgery (preoperative prednisolone plus NSAID group). Comparison groups received a combination of corticosteroid and NSAID drops initiated on the day of surgery (postoperative prednisolone plus NSAID group), NSAID eye drop monotherapy initiated 3 days before surgery or on the day of surgery (preoperative NSAID and postoperative NSAID groups, respectively), and sub-Tenon depot of dexamethasone administered at the conclusion of surgery (sub-Tenon group). Participants in the sub-Tenon group were not given anti-inflammatory drops. Corticosteroid drops were prednisolone acetate, 1% (Pred Forte; Allergan); NSAID drops, ketorolac tromethamine, 0.5% (Acular; Allergan); and sub-Tenon depot, 0.5 mL of dexamethasone phosphate, 4 mg/mL (Dexamethasone Krka [Krka] or Dexavital [Vital Pharma Nordic]). All drops were administered 3 times per day until 3 weeks postoperatively, and adherence was monitored by asking participants, in an unstructured manner, if they were taking their drops; no formal recording of compliance was collected. Ethics committee approval was obtained from the Committee on Health Research Ethics, Capital Region, Denmark. The study was approved by the Danish Medicines Agency and the Danish Data Protection Agency. Accordance with the good clinical practice quality standard was monitored by the good clinical practice unit at Copenhagen University Hospital, Copenhagen, Denmark. The study was conducted in accordance with the Declaration of Helsinki,^15^ and all participants provided written informed consent. Participants received no incentives or compensation for participation. This study followed the Consolidated Standards of Reporting Trials (CONSORT) reporting guideline.

### Participants and Sample Size

**Figure 1.**
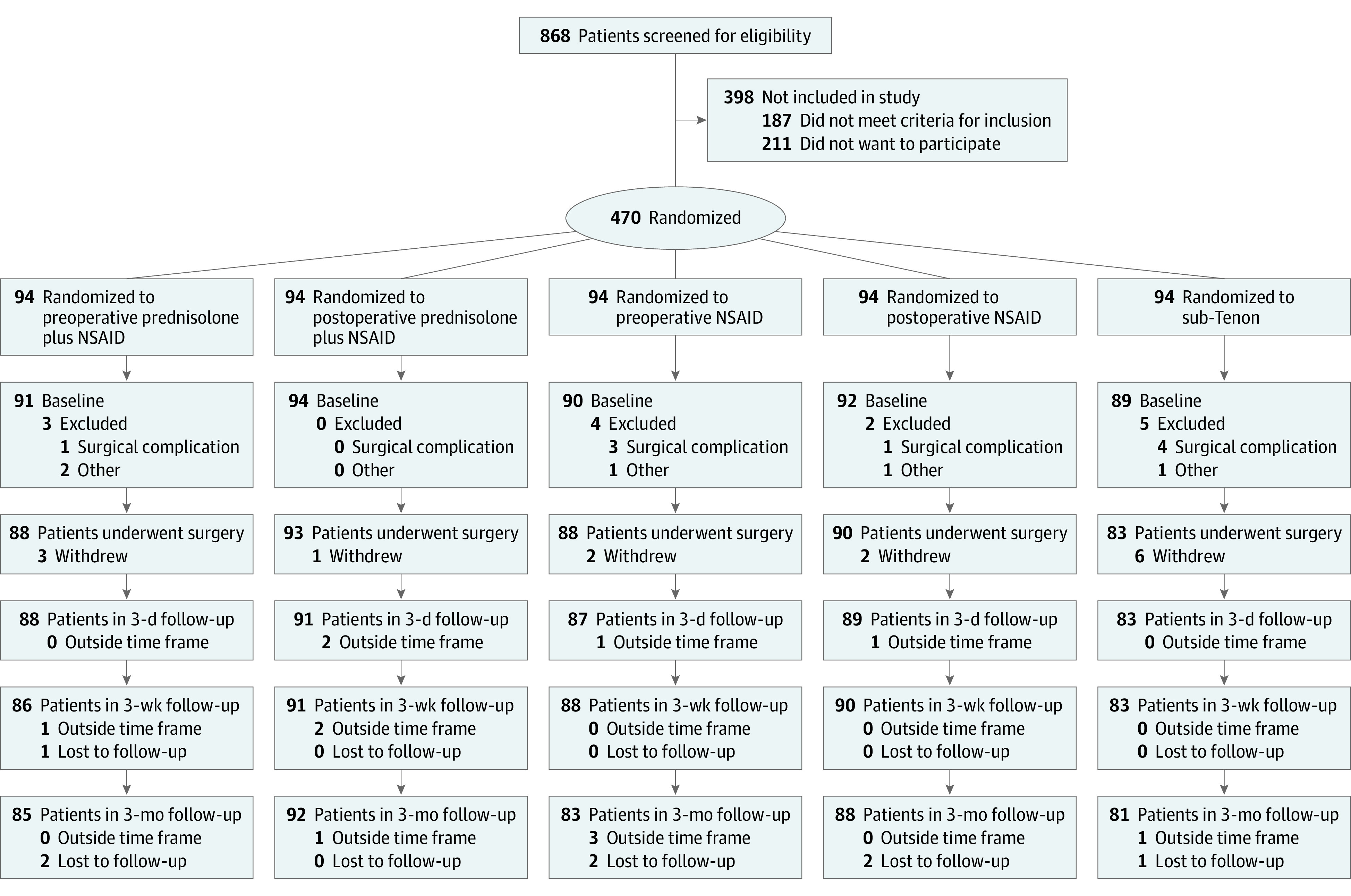
Consort Diagram for Flow of Participants Preoperative prednisolone plus nonsteroidal anti-inflammatory drug (NSAID) indicates group receiving a combination of prednisolone, 1%, and ketorolac tromethamine, 0.5%, eye drops 3 times per day until 3 weeks after surgery with initiation 3 days before surgery; postoperative prednisolone plus NSAID, group receiving a combination of prednisolone, 1%, and ketorolac, 0.5%, eye drops 3 times per day until 3 weeks after surgery with initiation on the day of surgery; preoperative NSAID, group receiving monotherapy with ketorolac, 0.5%, eye drops 3 times per day until 3 weeks after surgery with initiation 3 days before surgery; postoperative NSAID, group receiving monotherapy with ketorolac, 0.5%, eye drops 3 times per day until 3 weeks after surgery with initiation on the day of surgery; and sub-Tenon, group receiving sub-Tenon depot consisting of 0.5 mL of dexamethasone phosphate, 4 mg/mL. Participants who withdrew did so before surgery and before investigational medicine was administered; for those outside the time frame, postoperative visits took place outside the prespecified 2 to 4 days for the 3-day visit, 14 to 28 days for the 3-week visit, and 60 to 120 days for the 3-month visit. Some participants who attended a visit outside the specified time frame returned for the next postoperative visit within the specified time frame of that visit.

We recruited participants among patients scheduled for cataract surgery at the Department of Ophthalmology, Rigshospitalet Glostrup, Glostrup, Denmark, from February 1, 2018, to August 15, 2019. Inclusion criteria were age-related cataract, capacity to consent, and consent to participation. In addition, women had to be postmenopausal. Exclusion criteria were known allergy to any content of the pharmaceuticals used; medical history of retinal vein occlusion, epiretinal membrane, uveitis, glaucoma, retinal detachment, diabetes, exudative age-related macular degeneration, or age-related macular degeneration with geographical atrophy; and significant complications of surgery such as posterior capsule rupture/vitreous loss, choroidal hemorrhage, or dislocated lens material. Participants with complications were excluded when complications were detected (Figure 1). Sample size was set at 94 per interventional group, which allowed us to detect a difference of 5 μm in central retinal thickness with a power of 90% at a 5% significance level or a power of 80% at a 1.25% significance level. Five micrometers was chosen at the investigators’ discretion.

### Randomization

One eye per participant was included using a computerized coin toss to decide which eye to include if both eyes were eligible. Immediately after receiving signed consent for participation, participants were randomized to 1 of the 5 interventional groups (Figure 1) by the same examiner (J.H.E.). We used the randomization instrument in Research Electronic Data Capture (REDCap)^16,17^ hosted at Capital Region, Denmark. A block-randomized list was created at Sealed Envelope (https://www.sealedenvelope.com/simple-randomiser/v1/lists) and uploaded to REDCap by an independent researcher. The length of the list was 470, and block sizes were 5, 10, and 15 in random order.

### Surgical Procedure

All surgical procedures were performed by experienced surgeons (including L.M.H.), with experience defined as a minimum of 1000 cataract procedures performed within the 2 previous years. The surgical procedure was phacoemulsification with implantation of a hydrophobic intraocular lens (IOL) in the lens bag. Oxybuprocaine was used as local anesthetic, and eyes were instilled with phenylephrine hydrochloride, 10%, tropicamide, 1%, and ketorolac, 0.5%, before surgery. We used a 2.4-mm main incision and a 1-mm side port incision. Intracameral lidocaine hydrochloride, 1%, and viscoelasticum were instilled, followed by capsulorhexis and hydrodissection. For phacoemulsification, we used a surgical microscope and probe (Infinity Vision System; Alcon) with a miniflared 0.9-mm tip. On conclusion, the anterior chamber was irrigated with 1 mL of cefuroxime, 2.5 mg/mL.

### Examinations and Outcomes

Follow-up was completed December 18, 2019. Participants were examined at the preoperative visit (baseline) and 3 days, 3 weeks, and 3 months postoperatively. The primary outcome was central macular thickness at the 3-month visit measured by optical coherence tomography (DRI OCT Triton; Topcon Europe Medical BV) using the central 1.0-mm zone (central subfield thickness [CST]) of the ETDRS (Early Treatment Diabetic Retinopathy Study) grid obtained from the built-in software (IMAGEnet 6; Topcon Europe Medical BV).^18,19^ Secondary outcomes were CST at 3 weeks postoperatively, corrected distance visual acuity (CDVA) in logarithm to the minimal angle of resolution (logMAR) using an ETDRS chart, intraocular pressure (IOP), and subjective tolerance of treatment. Intraocular pressure was measured using a rebound tonometer (iCare Finland) and controlled by Goldmann applanation tonometry if the IOP was greater than 25 mm Hg. Subjective tolerance to treatment was assessed 3 days and 3 weeks postoperatively by asking the participants if they experienced no discomfort or mild discomfort or moderate or severe discomfort. Cataract severity was graded using the Age-Related Eye Disease Study classification.^20^ Adverse events were defined as events that were not expected as part of routine surgery for cataract or baseline conditions or as events that led to additional treatment. Corneal edema or dryness were not noted as adverse events unless additional treatment was initiated. The decision to institute additional treatment was made by experienced physicians (including J.H.E., L.M.H., and L.K.). Any need for additional topical anti-inflammatory, lubricating, IOP-lowering, or antibiotic treatment was registered. Adverse events with relation to the eyes were grouped as pain/soreness, insufficiently controlled anterior chamber inflammation, cystoid abnormalities on optical coherence tomography, dryness, corneal abrasion, swollen/red externa, elevated IOP of greater than 25 mm Hg, significant corneal edema, or other.

### Statistical Analysis

Data were analyzed from February 17 to June 15, 2020. All statistical analyses were conducted according to a prespecified analytical plan using the R statistical software, version 3.6.0 (R Program for Statistical Computing)^21^ and the nlme package (R Program for Statistical Computing). Pairwise comparisons between the control and comparison groups used a constrained linear mixed model with an unstructured covariance pattern and inherent baseline adjustment.^22^ Dichotomous outcomes were analyzed with Fisher exact test. All hypothesis tests were 2 sided. Primary analyses (change in CST 3 months postoperatively, relative to the control group) were adjusted for multiple testing using Bonferroni correction, resulting in a significance level of .0125. All secondary analyses were corrected for multiple testing using a false discovery rate correction.^23^ We considered an adjusted *P* < .05 to indicate statistical significance. Because some participants were excluded after allocation, analyses followed a modified intention-to-treat approach that included all participants who provided baseline data. The constrained linear mixed model implicitly handled missing data by maximum likelihood estimation. We evaluated the sensitivity of our analyses (eTables 1-6 in Supplement 3). An independent researcher masked statistical analyses by renaming the interventional groups. Full masking was not possible because sub-Tenon treatment was recognizable for participants and outcome assessors. After unmasking, we made analyses of the combination of corticosteroid and NSAID eye drops vs NSAID monotherapy and analyses of preoperative vs postoperative initiation of eye drop therapy by pooling relevant groups. As an exploratory post hoc analysis, we counted cases with a CST increase of at least 10%.

## Results

A total of 470 participants (mean [SD] age, 72.2 [7.0] years; 290 women [61.7%] and 180 men [38.3%]) were included and randomized, with 94 participants in each interventional group (Table 1). After randomization, 14 participants were excluded. Nine of these were excluded owing to surgical complications and 5 were excluded owing to other criteria (eTable 7 in Supplement 3); thus, 456 participants provided baseline data and 429 completed 3 months of follow-up (Figure 1).

**Table 1.** Baseline Characteristics of Included Participants and Use of Phacoemulsification Energy^a^

Characteristic	All participants (N = 470)	Treatment group				
		Preoperative prednisolone plus NSAID (n = 94)	Postoperative prednisolone plus NSAID (n = 94	Preoperative NSAID (n = 94)	Postoperative NSAID (n = 94)	Sub-Tenon (n = 94)
Sex, No. (%)						
Female	290 (61.7)	56 (60.0)	58 (61.7)	69 (73.4)	58 (61.7)	49 (52.1)
Male	180 (38.3)	38 (40.0)	36 (38.3)	25 (26.6)	36 (38.3)	45 (47.9)
Age, mean (SD), y	72.2 (7.0)	72.6 (7.1)	72.3 (7.0)	71.8 (7.0)	72.2 (7.0)	71.8 (7.1)
CST, mean (SD), μm	243.2 (21.8)	242.7 (19.4)	238.8 (20.5)	244.1 (23.7)	244.9 (24.3)	245.4 (20.4)
CDVA, mean (SD), logMAR^b^	0.29 (0.15)	0.29 (0.19)	0.31 (0.15)	0.29 (0.16)	0.27 (0.14)	0.29 (0.13)
IOP, mean (SD), mm Hg	14.3 (3.9)	13.9 (3.7)	14.3 (4.1)	14.6 (4.0)	14.3 (3.8)	14.2 (4.0)
AREDS, median (range)^c^	2.0 (<1.0 to >3.0)	2.5 (1.0 to >3.0)	2.0 (1.0 to >3.0)	2.0 (<1.0 to 3.0)	2.0 (<1.0 to >3.0)	2.0 (1.0 to 3.0)
CDE, median (IQR)^d^	7.9 (5.5 to 11.3)	9.2 (6.3 to 14.1)	8.3 (6.1 to 11.4)	7.4 (5.2 to 10.8)	7.8 (5.7 to 10.6)	7.1 (4.6 to 11.1)

Abbreviations: AREDS, Age-Related Eye Disease Study; CDE, cumulative dissipated energy; CDVA, corrected distance visual acuity; CST, central subfield thickness; IOP, intraocular pressure; IQR, interquartile range; logMAR, logarithm to the minimal angle of resolution; NSAID, nonsteroidal anti-inflammatory drug.

^a^ Baseline demographics were obtained from the preoperative evaluation; CDE, after cataract surgery. The preoperative prednisolone plus NSAID group served as control group. Sex of participants was obtained from the Danish central person registry. Groups are described in the legend to Figure 1.

^b^ The Snellen equivalent to the baseline mean CDVA is approximately 20/40.

^c^ Indicates AREDS classification of cataract score for nuclear cataract.

^d^ Measured as units of phacoemulsification energy.

### Central Subfield Thickness

**Figure 2.**
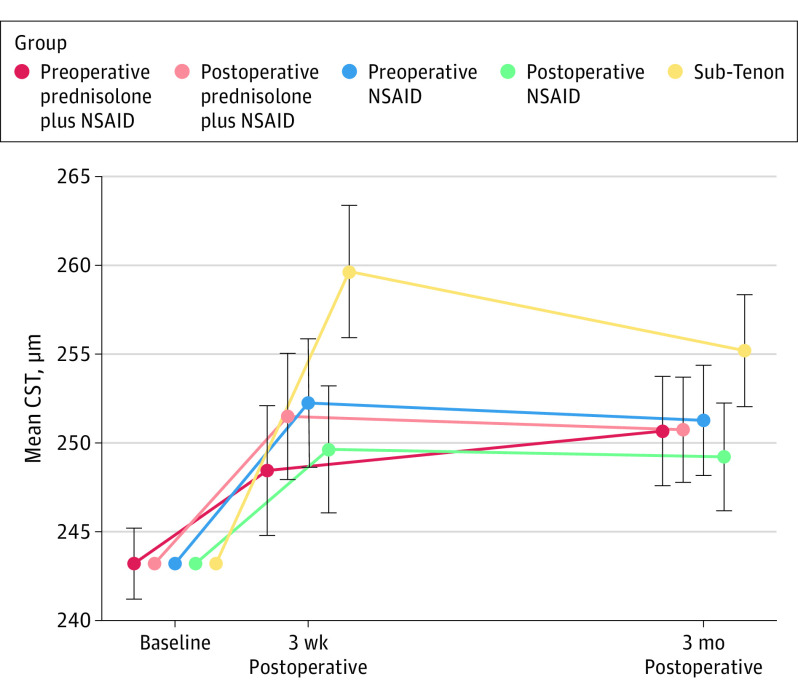
Mean Central Subfield Thickness (CST) From Baseline to 3 Months After Surgery Error bars represent 95% CIs. Only 1 error bar is presented at baseline because estimates per definition are the same for all groups in the constrained linear mixed model with inherent baseline adjustment. Groups are described in the legend for Figure 1.

Three months after surgery, the mean CST was 250.7 (95% CI, 247.6-253.7) μm in the preoperative prednisolone plus NSAID group, 250.7 (95% CI, 247.8-253.7) μm in the postoperative prednisolone plus NSAID group, 251.3 (95% CI, 248.2-254.4) μm in the preoperative NSAID group, 249.2 (95% CI, 246.2-252.3) μm in the postoperative NSAID group, and 255.2 (95% CI, 252.0-258.3) μm in the sub-Tenon group. Differences between the control (preoperative prednisolone plus NSAID) and the comparison groups were 0.1 (98.75% CI, −5.4 to 5.5) μm (*P* = .97) with the postoperative prednisolone plus NSAID group, 0.6 (98.75% CI, −5.0 to 6.2) μm (*P* = .79) with the preoperative NSAID group, −1.5 (98.75% −7.0 to 4.1) μm (*P* = .51) with the postoperative NSAID group, and 4.5 (98.75% CI, −1.1 to 10.1) μm (*P* = .04) with the sub-Tenon group. Results are presented in Figure 2 and Table 2. The numbers of participants with CST increases of at least 10% are presented in eTable 8 in Supplement 3.

**Table 2.** Results From Analyses of CST, IOP, and Visual Acuity^a^

Measurement	Patient group				
	Preoperative prednisolone plus NSAID^b^	Postoperative prednisolone plus NSAID^c^	Preoperative NSAID^c^	Postoperative NSAID^c^	Sub-Tenon^c^
CST, μm					
Baseline^d^	243.2 (241.2 to 245.2)	NA	NA	NA	NA
3-wk Change	5.2 (1.6 to 8.9)	3.0 (−2.0 to 8.1)	3.8 (−1.3 to 8.9)	1.2 (−3.9 to 6.3)	11.2 (6.0 to 16.4)
*P* value	NA	.24	.14	.65	<.001
Adjusted *P* value^e^	NA	.48	.32	.91	<.001
3-mo Change	7.5 (4.4 to 10.5)	0.1 (−5.4 to 5.5)^f^	0.6 (−5.0 to 6.2)^f^	−1.5 (−7.0 to 4.1)^f^	4.5 (−1.1 to 10.1)^f^
*P* value	NA	.97	.79	.51	.04
IOP, mm Hg					
Baseline^d^	14.3 (13.9 to 14.6)	NA	NA	NA	NA
3-d Change	−0.7 (−1.3 to −0.1)	−0.2 (−1.0 to 0.7)	−2.1 (−2.9 to −1.2)	−2.5 (−3.4 to −1.7)	−3.2 (−4.1 to −2.4)
*P* value	NA	.68	<.001	<.001	<.001
Adjusted *P* value^e^	NA	.92	<.001	<.001	<.001
3-wk Change	−2.2 (−2.7 to −1.6)	0.3 (−0.4 to 1.0)	−1.1 (−1.8 to −0.4)	−1.5 (−2.1 to −0.8)	−1.0 (−1.7 to −0.3)
*P* value	NA	.36	.001	<.001	.004
Adjusted *P* value^e^	NA	.65	.004	<.001	.01
3-mo Change	−3.3 (−3.8 to −2.8)	0.2 (−0.5 to 0.8)	−0.3 (−0.9 to 0.4)	−0.3 (−0.9 to 0.3)	−0.2 (−0.8 to 0.5)
*P* value	NA	.57	.42	.36	.65
Adjusted *P* value^e^	NA	.87	.71	.65	.91
CDVA, logMAR^g^					
Baseline^d^	0.29 (0.28 to 0.30)	NA	NA	NA	NA
3-d Change	−0.19 (−0.22 to −0.16)	−0.01 (−0.05 to 0.04)	−0.03 (−0.08 to 0.01)	0.00 (−0.05 to 0.04)	.01 (−0.04 to 0.05)
*P* value	NA	.80	.14	.86	.71
Adjusted *P* value^e^	NA	>0.99	.31	>.99	.95
3-wk Change	−0.27 (−0.30 to −0.24)	0.01 (−0.02 to 0.04)	0.00 (−0.03 to 0.03)	0.00 (−0.04 to 0.03)	0.03 (0.00 to 0.07)
*P* value	NA	.56	.95	.80	.06
Adjusted *P* value^e^	NA	.84	>.99	>.99	.16
3-mo Change	−0.30 (−0.33 to −0.28)	0.01 (−0.02 to 0.04)	0.01 (−0.02 to 0.04)	0.00 (−0.03 to 0.03)	0.02 (−0.01 to 0.05)
*P* value	NA	.53	.48	.97	.18
Adjusted *P* value^e^	NA	.81	.78	>.99	.38

Abbreviations: CDVA, corrected distance visual acuity; CST, central subfield thickness; IOP, intraocular pressure; NA, not applicable; NSAID, nonsteroidal anti-inflammatory drug.

^a^ All estimates were derived from the constrained linear mixed model with inherent baseline adjustment.

^b^ Presented as mean change from baseline (95% CI).

^c^ Unless otherwise indicated, data are presented as differences from the preoperative prednisolone plus NSAID group as mean (95% CI).

^d^ The baseline value was the same for all groups.

^e^ Adjusted for false discovery rate.

^f^ Presented as mean (98.75% CI) difference to account for Bonferroni correction.

^g^ For reference, the approximate Snellen equivalents to logMAR are as follows: logMAR = 0.3 is 20/40; logMAR = 0.1 is 20/25; and logMAR = 0.0 is 20/20.

### IOP and Visual Acuity

The mean IOP decreased in all groups postoperatively, but it was lower in groups not receiving prednisolone in the early postoperative period. At 3 months, we found no differences compared with the preoperative prednisolone plus NSAID group (−3.3 [95% CI, −3.8 to −2.8] mm Hg) in the postoperative prednisolone plus NSAID group (0.2 [95% CI, −0.5 to 0.8] mm Hg), the preoperative NSAID group (−0.3 [95% CI, −0.9 to 0.4] mm Hg), the postoperative NSAID group (−0.3 [95% CI, −0.9 to 0.3] mm Hg), and the sub-Tenon group (−0.2 [95% CI, −0.8 to 0.5] mm Hg (Table 2 and the eFigure in Supplement 3). Corrected distance visual acuity improved in all groups 3 months postoperatively with no identified differences compared with preoperative prednisolone plus NSAID group (Table 2).

### Subjective Tolerance

Moderate or severe discomfort was reported by a total of 32 participants (7.3%) at 3 days postoperatively and a total of 13 (3.0%) at 3 weeks postoperatively. We identified no differences compared with the preoperative prednisolone plus NSAID group (Table 3).

**Table 3.** Subjective Tolerance, Adverse Events, Added Treatment, and Extra Visits

Outcome	Patient group^a^				
	Preoperative prednisolone plus NSAID	Postoperative prednisolone plus NSAID	Preoperative NSAID	Postoperative NSAID	Sub-Tenon
Subjective tolerance, No./total No. (%)					
3-d Postoperatively					
No or mild discomfort	81/88 (92.0)	87/93 (93.5)	82/87 (94.3)	87/90 (96.7)	72/83 (86.7)
Moderate or severe discomfort	7/88 (8.0)	6/93 (6.5)	5/87 (5.7)	3/90 (3.3)	11/83 (13.3)
3-wk Postoperatively					
No or mild discomfort	86/87 (98.9)	92/93 (98.9)	85/88 (96.6)	86/90 (95.6)	79/83 (95.2)
Moderate or severe discomfort	1/87 (1.2)	1/93 (1.1)	3/88 (3.4)	4/90 (4.4)	4/83 (4.8)
Adverse events, No./total No. (%)					
Total	23/88 (26.1)	32/93 (34.4)	27/88 (30.7)	28/90 (31.1)	65/83 (78.3)^b^
Pain/soreness	2/88 (2.3)	2/93 (2.2)	1/88 (1.1)	0	22/83 (26.5)^b^
Insufficiently controlled inflammation	2/88 (2.3)	5/93 (5.4)	5/88 (5.7)	4/90 (4.4)	40/83 (48.2)^b^
Cystoid abnormalities on OCT	2/88 (2.3)	3/93 (3.2)	4/88 (4.5)	0	11/83 (13.3)^c^
Dryness	7/88 (8.0)	18/93 (19.4)	13/88 (14.8)	14/90 (15.6)	23/83 (27.7)^d^
Corneal abrasion	3/88 (3.4)	1/93 (1.1)	0	4/90 (4.4)	3/83 (3.6)
Swollen/red externa	2/88 (2.3)	0	1/88 (1.1)	1/90 (1.1)	1/83 (1.2)
IOP >25 mm Hg	0	1/93 (1.1)	0	1/90 (1.1)	0
Corneal edema	4/88 (4.5)	3/93 (3.2)	1/88 (1.1)	2/90 (2.2)	4/83 (4.8)
Other	7/88 (8.0)	4/93 (4.3)	9/88 (10.2)	7/90 (7.8)	9/83 (10.8)
Added treatment, No./total No. (%)					
Anti-inflammatory	8/88 (9.1)	10/93 (10.8)	12/88 (13.6)	5/90 (5.6)	47/83 (56.6)^b^
Lubricating	8/88 (9.1)	20/93 (21.5)	15/88 (17.0)	14/90 (15.6)	26/83 (31.3)^d^
IOP lowering	0	1/93 (1.1)	0	1/90 (1.1)	0
Topical antibiotic	3/88 (3.4)	1/93 (1.1)	1/88 (1.1)	5/90 (5.6)	2/83 (2.4)
Extra visits					
No. of participants	8	9	15	10	27^b^
No. of extra visits	14	16	17	12	33^d^

Abbreviations: IOP, intraocular pressure; NSAID, nonsteroidal anti-inflammatory drug; OCT, optical coherence tomography.

^a^ Adverse events, added treatment, and extra visits are totals for the entire study period. As standard, participants with need for additional anti-inflammatory treatment received combination of corticosteroid and NSAID eye drops 3 times per day until 3 weeks after surgery if the need emerged in the early postoperative period. If corticosteroid eye drops were already used, frequency was increased for a few days. Cystoid abnormalities on OCT were treated with corticosteroid and NSAID eye drops 3 times per day for 4 weeks followed by a clinical control. Total adverse events refers to the number of participants with 1 or more adverse events. *P* values were adjusted with false discovery rate method. Groups are described in the legend to Figure 1.

^b^ Adjusted *P* < .001.

^c^ Adjusted *P* < .05.

^d^ Adjusted *P* < .01.

### Adverse Events, Additional Treatment, and Unscheduled Visits

Adverse events and addition of anti-inflammatory treatment were registered more often in the sub-Tenon group, but no differences were found between the preoperative prednisolone plus NSAID group and the remaining groups. Two participants were treated for elevated IOP in the immediate postoperative period, but no IOP elevations of greater than 25 mm Hg were found at any postoperative visit. In the sub-Tenon group, there were more unscheduled visits, but no differences were found between the remaining groups and the preoperative prednisolone plus NSAID group. Results are presented in Table 3.

### NSAID Monotherapy vs Combination of Topical Corticosteroid and NSAID

No differences were identified between NSAID monotherapy and the combination of prednisolone and NSAID drops regarding CST or CDVA at any postoperative visit after pooling combination groups (preoperative and postoperative prednisolone plus NSAID) and NSAID monotherapy groups (preoperative and postoperative NSAID). Intraocular pressure was lower for NSAID monotherapy 3 days and 3 weeks postoperatively compared with the combination of prednisolone and NSAID. No differences in IOP were identified 3 months postoperatively (eTable 9 in Supplement 3).

### Preoperative vs Postoperative Initiation of Topical Treatment

We pooled groups with preoperative initiation (preoperative prednisolone plus NSAID and preoperative NSAID) and those with postoperative initiation (postoperative prednisolone plus NSAID and postoperative NSAID) of treatment. In this analysis, we identified no differences between preoperative or postoperative initiation of treatment for CST, CDVA, or IOP at any postoperative visit (eTable 10 in Supplement 3).

### Sensitivity of Results

We found that our overall conclusions were not sensitive to handling of missing values, to restriction to the per-protocol population, or to inclusion, exclusion, or truncation of extreme outliers. In addition, postrandomization confounding from sex, age, and phacoemulsification energy was unlikely (eTables 1-6 in Supplement 3). Characteristics of participants with and without complete data are presented in eTable 11 in Supplement 3.

## Discussion

We investigated the effect of anti-inflammatory prophylaxis on macular thickening after uncomplicated cataract surgery in a randomized clinical study that evaluated the additive effect of prednisolone to NSAID eye drops in a fashion that allowed us to compare the effect of initiating treatment preoperatively with initiation on the day of surgery. In addition, we evaluated the effect of dropless surgery using a sub-Tenon dexamethasone depot. We found that NSAID plus corticosteroid drops were not superior to NSAID monotherapy or sub-Tenon dexamethasone depot for postoperative central macular thickening after uncomplicated cataract surgery.

One of the primary purposes of prescribing an anti-inflammatory prophylactic treatment in parallel with cataract surgery is to prevent PCME. Pseudophakic cystoid macular edema is infrequent and heterogeneously defined, which makes it difficult to use the incidence as an outcome in clinical trials. Instead, the CST measured with optical coherence tomography is generally accepted as a measure for comparing efficacy of prophylactic regimens. We did not find differences in CST between groups receiving NSAID monotherapy or a combination of NSAID and prednisolone. A large European multicenter trial^19^ compared corticosteroid monotherapy (dexamethasone, 0.1%) with NSAID monotherapy (bromfenac, 0.09%) and a combination of both and found no significant differences between the combination of corticosteroid and NSAID and NSAID monotherapy, whereas dexamethasone monotherapy resulted in increased macular thickness 6 weeks postoperatively. However, NSAID monotherapy is still controversial for prophylactic treatment after cataract surgery, especially in the US,^24^ and NSAIDs are not recommended by the American Academy of Ophthalmology.^10^

A main argument for not recommending NSAIDs has been the lack of comparison with prednisolone drops, because they are theoretically more efficient than more potent corticosteroids owing to a greater intraocular bioavailability.^10^ Another argument was that the increased effect of adding NSAIDs to corticosteroids was likely caused by increased dosing. To address the first argument, we chose prednisolone rather than more potent corticosteroid drops for the combination groups. Our results showed that NSAID monotherapy was comparable to combined prednisolone and NSAID drops, even though the total dose was higher in the combination groups. This outcome corroborates the conclusions from other randomized trials that compared NSAID monotherapy with combinations of corticosteroid and NSAID eye drops; namely, there is no significant additional effect of adding topical corticosteroids to topical NSAIDs parallel to uncomplicated cataract surgery.^19,25^ It could be argued that a result of no statistical significance is not evidence that there is no difference between groups. However, our study was powered to detect differences between groups as small as 5 μm, which we expected to be within normal variation between 2 measurements. Differences in CST and CDVA between eye drop groups and controls were minimal, and CIs were narrow. Therefore, it is unlikely that a larger sample size would uncover any clinically relevant differences.

Preoperative initiation of therapy did not result in decreased CST compared with postoperative initiation, even after pooling relevant groups. A previous randomized clinical trial^9^ concluded that preoperative initiation of ketorolac, 0.4%, was advantageous in the early postoperative period but showed no significant effects on long-term outcomes. No data on central macular thickness were reported, and the groups were small. We found no effect of preoperative initiation on any outcome measures.^26^

In the sub-Tenon group, CST was significantly increased 3 weeks postoperatively compared with the preoperative prednisolone plus NSAID group, but the difference was no longer significant 3 months after surgery. More than half of the participants in the sub-Tenon group required additional topical anti-inflammatory treatment during the study period, which might explain why no difference in CST was found at 3 months. Other randomized^13^ and nonrandomized^2,27^ studies have found comparable results between dropless surgery and corticosteroid eye drop monotherapy, but we were unable to locate reports comparing dropless surgery with combined corticosteroid and NSAID eye drops or NSAID monotherapy. We used a sub-Tenon depot of a highly potent corticosteroid, dexamethasone phosphate, and refrained from using triamcinolone, which has a much longer period of action, because of fear of sustained IOP elevations.^14^ Although it was safe to administer the sub-Tenon dexamethasone depot, it was not comparable to NSAID or prednisolone plus NSAID in preventing macular thickening after cataract surgery. In patients with suspected poor adherence with topical treatment, placing a depot of corticosteroid during surgery could serve as adjuvant therapy. However, IOP should be monitored regularly if triamcinolone is used.^28^

Intraocular pressure was lower during eye drop treatment in groups who did not use corticosteroid drops compared with those who did. However, the mean IOP in all groups was low in the reference range, and there were no elevations of greater than 25 mm Hg at any postoperative visit. There were no differences in visual acuity at any time point, but adverse events and unscheduled visits were more often encountered in the sub-Tenon group.

### Limitations

This study has some limitations. We used a modified intention-to-treat approach for our analyses because some participants were excluded after allocation. We included patients with an expected low risk of complications, including macular edema, after cataract surgery, and the surgeons were experienced. Thus, our results may only apply to such patients and not patients with risk factors (eg, diabetes).^28,29^ The study did not include a corticosteroid eye drop monotherapy group and therefore did not compare corticosteroid drops with NSAID drops. Prednisolone, 1%, is less potent than other commonly used corticosteroid drops. Other approaches to dropless surgery exist that may prove more efficient than sub-Tenon dexamethasone depot as used in this study.^30,31^ Follow-up ended 3 months after surgery, and later presentations of PCME may not have been identified. The decision to add anti-inflammatory treatment was made by an unmasked physician.

## Conclusions

In this randomized clinical trial, we found that a combination of prednisolone and NSAID eye drops was not superior to NSAID monotherapy and that initiating prophylactic treatment 3 days before surgery was not superior to initiating treatment on the day of surgery in preventing thickening of the central subfield after uncomplicated cataract surgery. In addition, we found that dropless surgery with a sub-Tenon dexamethasone depot was inferior to a combination of prednisolone and NSAID eye drops and that anti-inflammatory eye drops were required in a substantial number of participants in the sub-Tenon dexamethasone depot group. Intraocular pressure was higher in groups that used prednisolone eye drops compared with NSAID monotherapy and sub-Tenon dexamethasone depot in the first 3 weeks postoperatively. Therefore, NSAID monotherapy with initiation on the day of surgery may be preferred as an anti-inflammatory prophylactic regimen in uncomplicated cataract surgery.

## Notes

Supplement 1.
Trial Protocol

Supplement 2.
Statistical Analysis Plan

Supplement 3. **eFigure.** Intraocular Pressure From Baseline to 3 Months After Surgery **eTable 1.** Best Case Control Analyses **eTable 2.** Worst Case Control Analyses **eTable 3.** Per-Protocol Analyses **eTable 4.** Analyses Without Extreme Outliers **eTable 5.** Analyses With Truncated Extreme Outliers **eTable 6.** Analyses for Postrandomization Confounding **eTable 7.** Reasons for Exclusion **eTable 8.** Number of Participants With CST Increase ≥10% **eTable 9.** Combination vs NSAID Monotherapy **eTable 10.** Preoperative vs Postoperative Initiation of Prophylactic Treatment **eTable 11.** Baseline Characteristics for Completers, Noncompleters, and Drop-outs

Supplement 4.
Data Sharing Statement
