# Supplementary material for: Prednisolone and Ketorolac vs Ketorolac Monotherapy or Sub-Tenon Prophylaxis for Macular Thickening in Cataract Surgery: A Randomized Clinical Trial
Source: JAMA Ophthalmol. 2021 Aug 12;139(10):1062–70. doi: 10.1001/jamaophthalmol.2021.2976 (PMC8529413; doi:10.1001/jamaophthalmol.2021.2976)
Supplement: Supplement 1. — Trial Protocol [file jamaophthalmol-e212976-s001.pdf]

Protocol

Effect of drop-less surgery compared to topical NSAID alone and combination of steroid and NSAID on central macular thickness after cataract surgery, a randomized controlled trial (Study for optimizing anti-inflammatory prophylaxis, “SOAP”)

Version 5

Updated 15 December 2017

EudraCT nr.: 2017-002666-47

Sponsor-investigator

Line Kessel

Consultant ophthalmologist, Associate Professor, MD, Ph.D., FEBO

Department of Ophthalmology, Rigshospitalet-Glostrup

Valdemar Hansens Vej 3, 2600 Glostrup

line.kessel.01@regionh.dk

Phone: +45 3863 4132

Collaborators

Glostrup Apotek

/Kristian Østergaard

Hovedvejen 101, 2600 Glostrup

glostrup@apotekeren.dk

Phone: +45 4396 0020

Signature

Line Kessel

Consultant ophthalmologist, Associate Professor, MD, Ph.D., FEBO

List of contents

List of abbreviations 4

Purpose 5

Introduction 5

- Control of the inflammatory response 5
- Routes of administration 6
- Timing of anti-inflammatory prophylaxis to surgery 6
- NSAID 6
- Steroids 7
- Advantages and disadvantages for steroids and NSAIDs 9

Methods 10

- Randomization 10
- Blinding 11

Participants 11

- Criteria for inclusion 11
- Criteria for exclusion 11
- Procedures for recruiting participants 12

Sample size and data analyses 12

Examinations and procedures 13

- Preoperative evaluation 13
- Surgical procedures 14
- 3rd day post-operative review 14
- 3-week post-operative review 14
- 3-month post-operative review 14

Outcomes 15

- Primary outcome: 15
- Secondary outcomes: 15

Background literature search 15

Risks, side effects and inconveniences 16

Registration of adverse effects 16

- Adverse effects and reactions 17
- Reporting adverse effects to the authorities 17
- Criteria for aborting the study 18

Ethical considerations 18
Time-schedule 19
Quality assurance 19
Data storage after the study has completed 20
Economy 21
References 22

List of abbreviations

PCME: pseudophakic macular edema
IOP: intraocular pressure
Steroid: glucocorticoid
NSAID: non-steroid anti-inflammatory drug
COX: cyclooxygenase
PGE_2: prostaglandin E_2
AAO: American Academy of Ophthalmologists
CMT: central macular thickness
OCT: optical coherence tomography
AMD: age-related macular degeneration
BCVA: best corrected visual acuity
IOL: Intraocular lens
RNFL: retinal nerve fiber layer
logMAR: logarithm to the minimal angle of resolution
CRF: Case Report Form
VFQ25: Visual Function Questionnaire-25
NIKE: Nationell Indikationsmodell för Kataraktextraktion

Purpose

The purpose of this study is to investigate which anti-inflammatory treatment is best at preventing postoperative inflammation following cataract surgery. We want to compare topical prophylaxis with NSAID eye drops to topical prophylaxis with a combination of NSAID and prednisolone. We also want to compare topical prophylaxis with eye drops to drop-less surgery where the anti-inflammatory drug is administered to the subconjunctival space at the conclusion of the surgical procedure. In addition, we want to investigate if topical anti-inflammatory prophylaxis should be initiated preoperatively or postoperatively. The primary outcome is change in central macular thickness, measured by optical coherence tomography, 3 months after surgery.

Introduction

Cataract surgery with extraction of the lens and implantation of an artificial intraocular lens is one of the most commonly performed surgical procedures in most westernized countries. It is a relatively safe procedure with a low rate of complications, but complications can be significant if they occur.[1] In the absence of other eye diseases, perfect vision is a realistic expectation after cataract surgery. Pseudophakic macular edema (PCME) is the most common cause of unexpected poor vision after cataract surgery affecting about 4 % - 25 % of patients even if prophylactic treatment is given.[2] With at least 50,000 cataract operations in Denmark per year a great number of patients are affected and even more will be in the future as the need for cataract surgery is expected to increase because of changing demographics.[3]

PCME is believed to be due to inflammation following cataract extraction. During the surgical process, the anterior segment of the eye is manipulated and lens proteins are liberated from the lens. This induces an inflammatory reaction with release of inflammatory mediators such as prostaglandins that are responsible for degradation of the blood-ocular barrier and leukocyte migration.[4] PCME is generally thought of as a self-limiting condition but large studies have shown that visual acuity remains decreased to below driver's license level in more than half of affected patients six months after surgery[5]. In some cases, the edema becomes chronic and results in permanently decreased vision. If the patient has a comorbidity such as diabetes mellitus or uveitis, the risk of developing PCME is higher due to the preexisting impairment of the blood-ocular barrier.[6,7]

Control of the inflammatory response

To minimize postoperative inflammation cataract surgeons usually prescribe prophylactic anti-inflammatory eye drops. Generally, two types are available: eye drops containing glucocorticoid (*"steroid"*) or non-steroidal anti-inflammatory drug (NSAID). Traditionally, steroid eye drops are favored but recent meta-analyses find that NSAIDs are more effective than steroid eye drops in controlling postoperative inflammation and reducing the risk for developing PCME, and suggests that NSAIDs should always be part of the postoperative anti-inflammatory prophylaxis.[2,8] The superior effect of NSAIDs theoretically may be due to increased penetrance to the retina compared to steroids. Both steroids and NSAIDs possess advantages and disadvantages which will be further described below.

Some investigators have proposed that a synergistic effect of steroid and NSAID treatment exists. However, others argue that such an effect would be unlikely due to the overlapping actions of NSAIDs and steroids[9] and there are no published studies evaluating if combination treatment is better than NSAID eye drops alone at preventing postoperative inflammation and PCME.

Routes of administration

We want to compare two different approaches for anti-inflammatory prophylaxis following cataract surgery, topical eye drop prophylaxis and "drop-less surgery". In topical prophylaxis eye drops must be applied several times a day for several weeks and in drop-less surgery a depot of steroid is administered once at the conclusion of the surgical procedure.

Administration of eye drops is challenging to many cataract patients who may not have the capacity to administer eye drops correctly because of dementia, arthritis etc.[10] A study found that 21 % were always assisted in administering eye drops and 36 % were unable to use the eye drops as prescribed.[11] In Denmark it is common to receive assistance from local community nurses to administer the eye drops which adds to the total societal costs of cataract surgery. Generally, NSAID eye drops are administered less frequently than steroid eye drops and therefore reduce the need for assistance. Drop-less surgery eliminates the need for postoperative eye drop administration if the intraocular pressure (IOP) is not elevated beyond an acceptable level, usually 25 mmHg.

Timing of anti-inflammatory prophylaxis to surgery

When should the anti-inflammatory prophylaxis be instituted? A report by the American Association of Ophthalmologists suggests that treatment with NSAIDs should be initiated 3 days before surgery but real scientific evidence is missing.[9] Previous studies have investigated the effect of steroid and NSAID versus steroid alone on anti-inflammatory prophylaxis following cataract surgery. An ongoing study, www.ClinicalTrials.gov identifier: NCT01774474, is testing steroid alone versus NSAID alone versus combination of the two in non-diabetic individuals. But no studies have, to our knowledge, compared the effect of NSAID alone versus steroid and NSAID versus drop-less surgery in a design that also allowed for an evaluation of whether to start anti-inflammatory treatment pre- or postoperatively.

NSAID

NSAIDs are inhibitors of cyclooxygenase enzymes which catalyze the synthesis of prostaglandins. COX exists in three isoforms, COX-1, 2 and 3, of which COX-1 is constitutively expressed and COX-2 is inducible. COX-3 has an unclear mechanism of action and its' role in inflammation is not characterized.[12,13] COX enzymes are found in the endothelial cells, retinal pigment epithelial cells, Müller cells, amacrine cells and ganglion cells of the human retina.[14] NSAIDs have an effect on COX-1 and COX-2, but different NSAIDs have various effects on the COX subtypes although none of the NSAIDs used for ophthalmic indications are classified as selective inhibitors of either COX subtype. Ketorolac is the most effective inhibitor of COX-1 of ophthalmic formulations, whereas bromfenac and nepafenac are more effective at inhibiting COX-2. Nepafenac is a prodrug intraocularly metabolized into the more potent NSAID amfenac, thus functioning as a reservoir for amfenac. Nepafenac quickly reaches peak concentration, but because it needs to be

hydrolyzed by intraocular enzymes, its' active state amfenac takes a relatively long time to reach peak concentration(Table 1).[15] Ketorolac peaks at 57.5 ng/ml after 60 minutes and remains at this level for at least 4 hours after a single application.[15] Studies have shown that ketorolac was the most effective inhibitor of prostaglandin E_2 (PGE_2) in aqueous humour[16,17] and another study found that patients treated with ketorolac had significantly lower vitreous PGE_2 levels than patients treated with nepafenac.[18] PGE_2 level is used as a marker for blood-retina- and blood-aqueous-barrier breakdown and is thought to reflect efficacy of PCME prophylaxis.[18] Ketorolac reaches the highest vitreous concentration compared to bromfenac and nepafenac, sufficient to effectively decrease vitreous PGE_2 levels and pseudophakic eyes reaches significantly higher vitreous levels than phakic eyes.[14,18]
Other aspects of deciding which NSAID to use include the price, level of discomfort when applying the drug and how easy it is for the patient to apply the eye drops. The cost of ketorolac is about half the price of both bromfenac and nepafenac and the experience at our institution is that ketorolac eye drops are less uncomfortable and easier to apply than nepafenac eye drops. When all the above mentioned information is taken into consideration, we find that ketorolac is the best choice of NSAID eye drops and that is the rationale behind choosing it for this study.

NSAID	Mean AUC (ng x h/ml)	C max (ng/ml)	T max (min)	COX-1 IC50 (µM)	COX-2 IC50 (µM)	Aqueous PGE2 (pg/ml)	Vitreous PGE2 (pg/ml)	Vitreous C (ng/ml)
bromfenac	47.2	25.9	240	0.0864	0.0112	288.7	247.2	0.96
ketorolac	176.9	57.5	60	0.0139	0.0911	224.8	189.6	2.8
nepafenac	308.9	205.3	30	82.3	>1000	320.4	267.7	1.1
amfenac	180.7	70.1	180	0.138	0.00177	–	–	2.0

Table 1: Pharmacokinetics and pharmacodynamics for different NSAIDs.[15,17,18] AUC = area under the curve, C max = peak concentration, T max = maximum time to reach peak concentration, IC50 = half-maximum inhibition concentration. PGE2-level = concentration of prostaglandin E2 after paracentesis or vitrectomy, vitreous C = vitreous concentration. AUC, C max, T max, COX-1 IC50 and COX-2 IC50 were measured after installation of a single drop of NSAID[15], PGE2-levels and vitreous concentration were measured after different regimes with several drops daily 1 or 3 days before surgery.[17,18] Concentration of ketorolac in the studies was either 0.4 % or 0.45 %, concentration of bromfenac was 0,09 % and concentration of nepafenac was 0,1 %

Steroids

Potency and penetrance to the aqueous humour vary between different steroids and the route of administration. To avoid systemic effects after cataract surgery, steroids are usually applied as eye drops, but can also be delivered as a single injection during surgery, referred to as "drop-less surgery". Clinical practice and reports in the literature have applied steroid injections to the subconjunctival and subtenonal space and as intracameral injections. [19–27]

To determine which steroid to use in the study, we went through the literature to find pharmacokinetic and pharmacodynamic data on steroid eye drops and injectable steroids used in human eyes. Our results are summarized in Table 2 and Table 3. Table 2 shows the relative anti-inflammatory potency and peak aqueous concentrations of different steroids when applied as a single drop. It is seen that the highly potent steroid dexamethasone has a much lower penetrance to the aqueous humor than prednisolone which, according to the American Academy of Ophthalmology, suggests that prednisolone eye drops have an advantage in preventing PCME compared to

dexamethasone eye drops.[9] Dexamethasone eye drops (Maxidex) are part of the standard prophylactic regimen at our institution, but based on the pharmacokinetic data and the recommendation by the AAO (that works as a world reference for best clinical practice) we prefer to test prednisolone eye drops in this study.

**Steroid**	**Anti-inflammatory potency**	**Aqueous C max (ng/ml)**	**Vitreous C max (ng/ml)**	**T max (min)**
Hydrocortisone	1	–	–	–
Prednisolone	4	669.9	–	120
Dexamethasone	30	31.0	1.1	91-120
Betamethasone	25	7.7	–	91-120

Table 2: Relative anti-inflammatory potency and peak aqueous concentration for steroid eye drops.[9,28–31] Aqueous C max was measured after a single eye drop. Vitreous C max was measured after a regime with 10 or 11 drops starting 1 day before vitrectomy. C max = peak concentration, T max = time to peak concentration. Concentration of prednisolone, dexamethasone and betamethasone eye drops were 1,0 %, 0,1 % and 0,1 % respectively.

Table 3 shows anti-inflammatory potency compared to cortisol and duration of action for injectable steroids. Longer duration of action may secure a long-term effect of the prophylaxis but on the other hand induces a risk for uncontrollable elevated IOP. Data on ocular penetrance were only found for dexamethasone, but suggest that dexamethasone may be effective at preventing inflammation and PCME since it reaches relatively high aqueous- and vitreous concentrations. Subconjunctival and subtenonal administration of steroid depots have both been found to be safe and effective regarding prophylaxis of intraocular inflammation.[22,26] Vascularization is more extended in the subconjunctival space compared to the subtenonal space which in theory means that a depot of steroid will wash out faster if placed subconjunctivally compared to subtenonally. The anti-inflammatory effect of a subtenonal steroid depot may therefore be slightly better than the anti-inflammatory effect of a subconjunctival steroid depot. In conclusion we find that subtenonal injection of dexamethasone may be the best alternative for anti-inflammatory prophylaxis after cataract surgery since it is highly potent, reaches high peak aqueous humour concentrations and may be less likely to increase the intraocular pressure than triamcinolone.[20,23,32] Tramcinolone is in a crystalloid form which gives it a longer duration of action than betamethasone and dexamethasone, 3-6 weeks compared to 1-10 days and 1-3 days respectively .[23]. In addition, when triamcinolone is injected directly into the anterior chamber the crystals will be visible, giving the pupil a milky-white appearance which may cause patients to be reluctant to undergo this prophylactic regimen. Subconjunctival betamethasone was shown to be a useful alternative to dexamethasone eye drops in prevention of PCME after cataract surgery.[26] Another study found subtenonal triamcinolone to be an effective and safe alternative to betamethasone eye drops in controlling intraocular inflammation after cataract surgery.[22] No studies have investigated the use of subtenonal or subconjunctival dexamethasone after cataract surgery but betamethasone and dexamethasone are equally potent[23] and both are more potent than triamcinolone. At our institution surgeons are experienced in administering subtenonal triamcinolone in the treatment of macular edema and subconjunctival dexamethasone following trabeculectomy, glaucoma drainage implant surgery, cyclodiode

laser and congenital glaucoma surgery. Hence dexamethasone is preferred over betamethasone. Subtenonal and subconjunctival administration of a steroid depot is reported to be safe.[22,26,33]

Steroid	Anti-inflammatory potency	Duration of action	Aqueous C max (ng/ml)	Vitreous C max (ng/ml)	T max (min)
Methylprednisolone	5	6-12 weeks or more	–	–	–
Triamcinolone	5	3-6 weeks or more	–	–	–
Betamethasone	25	1-10 days	–	–	–
Dexamethasone	25	1-3 days	858	72,5	150-180

Table 3: Relative anti-inflammatory potency compared to cortisol and duration of action after a single subconjunctival injection of different steroids.[23,32,34] C max = peak concentration, T max = time to peak concentration. The C max and T max values were estimated from a formula derived from study data. 0.5 ml of dexamethasone sodium phosphate 5 mg/ml was injected subconjunctivally, corresponding to a dose of 2.5 mg.[32]

Advantages and disadvantages for steroids and NSAIDs

Steroids have a wider anti-inflammatory effect than NSAIDs and the price is low. But treatment with steroids includes a risk for elevated intra ocular pressure, which is potentially harmful to the optic nerve if not successfully treated. Steroids particularly compose a risk to so called "steroid responders" who experience high elevations in IOP upon steroid treatment and to patients with glaucoma.[35] Additionally steroids are associated with impaired wound healing and increased risk of infections.[4]

Advantages for NSAIDs are the superior effect in preventing PCME compared to steroid eye drops[2] and the reduced need for eye drop application if only NSAID eye drops are needed. Complications to treatment with NSAID eye drops are mostly discomfort when the drop is applied, though corneal melts have been reported in rare cases.[36] A systematic review and meta-analysis did not find indications that the use of NSAID eye drops was associated with a higher risk of complications than steroid eye drops but found that IOP was significantly higher when steroid was used compared to NSAID.[2]

Methods

Study design

The study is a randomized controlled trial consisting of 5 interventional groups as shown in Figure 1. The participants who are allocated to the topical NSAID + prednisolone groups will serve as controls against the topical NSAID groups and the subtenonal dexamethasone group.

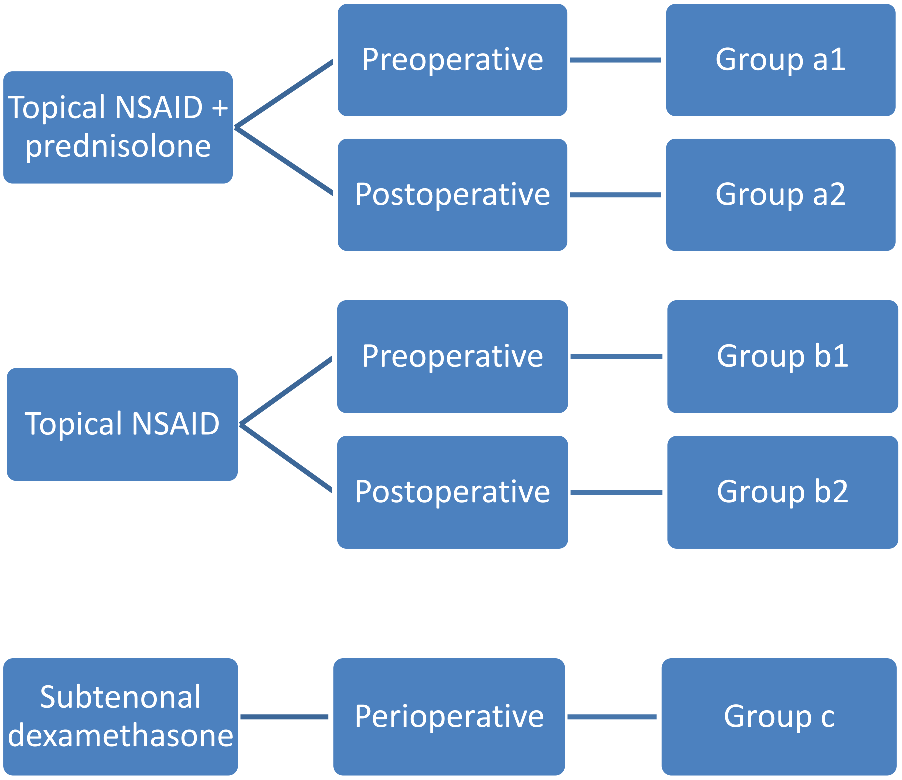

Figure 1: Schematic presentation of the interventional groups. Preoperative = treatment with eye drops is initiated 3 days prior to surgery; Postoperative = treatment with eye drops is initiated on the day of surgery. Perioperative = Subtenonal injection of dexamethasone is administered during surgery.

Randomization

Participants will be randomly assigned to 1 of the 5 interventional groups if they consent to participation in the study at the preoperative evaluation. Randomization will be done by a computerized algorithm. Participants allocated to group a1 and a2 will receive eye drop prophylaxis with both prednisolone (Pred Forte 1 %, prednisolone acetate, Allergan) and ketorolac (Acular 5 mg/ml, Allergan) both 3 drops per day for 3 weeks, participants allocated to group b1 and b2 will receive eye drop prophylaxis with ketorolac (Acular 5 mg/ml, Allergan) 3 drops per day for 3 weeks, and participants allocated to group c will receive a perioperative injection of dexamethasone dihydrogen phosphate (Dexamethason Krka 4 mg/ml,Krka) in the subtenonal space. The amount injected is 0.5 ml , equivalent to a dose of 2 mg. Acular is an off-the-shelf product, used as a standard for the purpose. Pred Forte and Dexamethason Krka are not marketed in Denmark but both are used widely in our neigbouring countries. Pred Forte will be provided via Glostrup

Apotek and Dexamethason Krka will be provided through the hospital pharmacy at Rigshospitalet-Glostrup, since a supply-agreement between the hospital and the manufacturer already exists. Pred Forte will be labelled according to the European rules on labelling of trial medicines.

Blinding

The study cannot be fully masked since the participants must be informed on the right treatment regime (whether to use no drops, one or two different kinds of drops daily) and the surgeon must be aware of whether to administer an injection of dexamethasone. Blinding will be secured by performing all statistical analyses blinded to randomization status.

Participants

Criteria for inclusion

- Patients with age-related cataracts
- Older than 18 years
- Women must be postmenopausal. Women are asked if they have menstruated within the preceding 12 months.
- Capacity to consent
- Scheduled to undergo cataract surgery at the ophthalmic department at Rigshospitalet-Glostrup, Denmark
- The surgeon must be experienced, defined by a minimum of 1000 cataract extractions completed
- Only 1 eye can be included for each participant, but there are no restrictions as to whether it is the eye that undergoes surgery first or last. If both eyes of a participant are eligible, it will be decided by randomization which eye to be included in the study
- Informed consent to participation

Criteria for exclusion

- Known allergy to any of the contents of the pharmaceuticals (active and in-active ingredients) used in the study
- Medical history of epiretinal membrane, retinal vein occlusion, retinal detachment, uveitis, glaucoma, diabetes mellitus, exudative age-related macular degeneration (AMD) or AMD with geographical atrophy
- Significant complications to surgery such as posterior capsule rupture/vitreous loss, choroidal hemorrhage, and dislocated lens material
- Pregnancy
- Fertile women, i.e. women who are not menopausal.
- Women who breastfeed

Procedures for recruiting participants

Written information about the study is sent to all patients referred to the cataract team for a preoperative visit during the inclusion period of the study, who may be asked for consent to participation. Whether a certain patient will receive written information or not will be decided in relation to booking of the preoperative visit. Thus, patients will have ample time to read the information and consider if they want to participate before they are seen at the preoperive visit. Referral to a preoperative visit is standard procedure for cataract patients and is not influenced by our study. Patients who fulfill the inclusion criteria and none of the exclusion criteria receive oral information about the study at the day of the pre-operative visit. The oral information is presented by a Ph.D.-student and not the principal investigator for practical reasons. The Ph.D.-student has taken part in designing the study and has completed the GCP-course offered by the GCP-unit. Oral information will be presented in a consultation room. The patient will be invited to bring a friend or relative to the preoperative visit and will be asked for consent after the oral information is given. If the patient consents to participation the consent can be withdrawn at any time and the patient will be treated according to the standard procedures for cataract surgery at Rigshospitalet-Glostrup. No blood samples will be taken.

Participation in the study will not restrict patients from taking other medications than the trial medication during the trial period. Hence participants may be treated with other medical products during the trial period.

Participants will not be offered any remuneration for participation.

Sample size and data analyses

To include enough participants to be able to detect a relevant difference in mean change in CMT between the interventional groups we performed a sample size calculation as shown in Table 4. The equation used for the calculations, when comparing two means from unpaired data is

$$n = \frac{(u+v)^2 \cdot (\sigma_1^2+\sigma_0^2)}{(\mu_1-\mu_0)^2}$$ [37]

Where n is the sample size, σ_1 and σ_0 are the standard deviations of the two groups and μ_1-μ_0 is the difference between the means. The values u and v are explained in the description of Table 4. We have decided that a clinically relevant minimum difference should be at least 5 micrometers (normal central retinal thickness is 250 microns). Setting the power of at least 90 %, we will need to include at least 84 participants in each interventional group. When adjusted for a 10 % dropout the minimal sample size is 94 participants and with 5 interventional groups the total study population will be 470 participants.

Data from the interventional groups will be compared to analyze topical prophylaxis vs drop-less surgery, topical NSAID alone vs topical NSAID and prednisolone, and preoperative initiation of topical prophylaxis vs postoperative initiation of topical prophylaxis.

Data on CMT, IOP, RNFL thickness, cells and flare count, and BCVA will be compared as differences in means. The mean change from baseline to 3 months postoperative is calculated for each interventional group and used for the comparisons as described above. Data on BCVA will be presented as the logarithm to the minimal angle of resolution (logMAR). The data analysis will be performed according to the principle of intention to treat.

The patient journal will be the data source for data on IOP, BCVA, cells and flare count and subjective patient tolerance of the prophylactic treatment. The OCT software will serve as data source for CMT and RNFL thickness. The Case Report Form (CRF) will not serve as data source.

**Power 90%**	**u = 1,28**	**v = 1,96**	**SD = 10**				
MIREDIF	1	2	3	4	5	6	7
n	2099,5	524,9	233,3	131,2	84,0	58,3	42,8
n adjusted	2332,8	583,2	259,2	145,8	93,3	64,8	47,6
n rounded	2333	584	260	146	94	65	48

**Power 80%**	**u = 0,84**	**v = 1,96**	**SD = 10**				
MIREDIF	1	2	3	4	5	6	7
n	1568,0	392,0	174,2	98,0	62,7	43,6	32,0
n adjusted	1742,2	435,6	193,6	108,9	69,7	48,4	35,6
n rounded	1743	436	194	109	70	49	36

Table 4: Sample size calculations. Based on data from a study that measured central macular thickness (CMT) by OCT before and 3 weeks after cataract surgery, ClinicalTrials.gov identification number NCT01686308. SD = standard deviation related to the change in mean CMT; u = one-sided percentage point of the normal distribution corresponding to 100 % - power; v = percentage point of the normal distribution corresponding to the two-sided significance level, in this case 5 %; MIREDIF = minimal relevant difference, the smallest difference we are able to detect at the given power, if it exists; µm = micrometer ("micron"); n = number of participants required to reach the given power; n adjusted = n adjusted for a 10 % drop-out; n rounded = number of participants required in practice.

Examinations and procedures

Preoperative evaluation

Participants will be examined preoperatively as they would if they were not included in the study. In addition, they will be examined with optical coherence tomography and laser flare photometry. The standard preoperative evaluation includes best corrected visual acuity, IOP measurement, evaluation of refractive errors, measurements of ocular biometry and IOL power calculation, and evaluation of the eye, including grading of the cataract by slit lamp imaging. Participants will be asked to fill out a questionnaires (Visual Function Questionnaire-25 (VFQ25) translated to Danish[38] and the Danish subjective complaints-scheme, part of the Nationell Indikationsmodell för Kataraktextraktion (NIKE)-score[39]) regarding their visual quality of life. Calculating the NIKE-score is standard procedure for preoperative visits.

Surgical procedures

All surgical procedures are performed in local anesthesia. Preoperatively, the eye is instilled with oxybuprocaine, phenylephrine 10%, tropicamide 1%, and ketorolac 0.5%. Then the eye is disinfected with povidone iodide 1% and chlorhexidine ethanol 0.5% and sterily draped. Main incision, 2.4 mm wide, and side-port incision, 1mm wide, are performed. Intracameral lidocain 1% and viscoelasticum (Healon OVD, Abbott Medical Optics) are instilled followed by capsulorhexis and hydrodissection. Ultrasound phacoemulsification is performed with the Alcon Infiniti machine using the mini-flared 0.9 mm tip. Residual cortex removal is done by co-axial irrigation and aspiration (I/A). Viscoelasticum is instilled again to inflate the bag where the IOL is placed. Then the viscoelastic is removed by I/A, the incisions are hydrated, and the anterior chamber is irrigated with 1 ml cefuroxime 2.5 mg/ml.

The surgical procedures for patients included in the study are the same as the surgical procedures for all patients with age-related cataracts at our institution.

3rd day post-operative review

As a standard, patients are reviewed at the hospital on the 1st to 3rd postoperative day. Study participants will be reviewed on the 3rd +/- 1 postoperative day. The standard postoperative review serves the purpose of detecting early postoperative complications and includes BCVA, IOP measurement and evaluation of the eye, including slit lamp imaging. Study participants will additionally undergo laser flare photometry to objectively evaluate inflammation in the anterior chamber. Compliance to the assigned prophylactic regime will be assessed by asking the participant.

3-week post-operative review

As part of the standard treatment, patients undergoing cataract surgery are reviewed 3 weeks postoperatively by a private ophthalmologist. Study participants will be reviewed 3 weeks +/- 4 days postoperatively at the department of ophthalmology and the review will include BCVA, IOP measurement, evaluation of refractive errors and biometry, OCT and evaluation of the eye, including slit lamp imaging. Compliance to the assigned prophylactic regime will be assessed by asking the participant.

3-month post-operative review

Standard treatment does not include a 3-month review, but study participants will be reviewed 3 months postoperatively to evaluate long term outcomes. The review must be 12 weeks +/- 2 weeks from the date of operation. The 3-month review will include BCVA, IOP-measurement, evaluation of refractive errors, OCT and evaluation of the eye, including slit lamp imaging. Participants will be asked to fill out the questionnaires VFQ25 and NIKE subjective complaints scheme.

All investigative procedures are non-invasive (except for the surgical procedure itself) and are not associated with any pain or discomfort.

Outcomes

Primary outcome:

- Change in central macular thickness (CMT) measured by optical coherence tomography 3 months after cataract surgery.

Secondary outcomes:

- Postoperative best corrected visual acuity in logarithm to the minimal angle of resolution (logMAR)
- Change in intraocular pressure (IOP)
- Optical nerve damage, assessed by measuring retinal nerve fiber layer (RNFL) by peripapillary optical coherence tomography (OCT)
- Flare and number of cells measured by laser flare-cell photometry
- Subjective patient tolerance of prophylactic treatment measured on a 4-point analog scale, where 0 = no discomfort, 1 = mild discomfort, 2 = moderate discomfort and 3 = severe discomfort.
- Patient related outcome measures obtained from questionnaire

All outcomes except subjective patient tolerance will be measured at baseline. CMT and RNFL will be measured again 3 weeks and 3 months postoperatively. BCVA and IOP will be measured at all visits. Subjective patient tolerance will be measured 3 days and 3 weeks postoperatively. Flare and number of cells will be measured 3 days postoperatively. Patient related outcome measures will be measured at 3 months postoperatively. Evaluation of optic nerve damage will be addressed by measuring the retinal nerve fiber layer and not by perimetry because the cataractous lens would confound the baseline results of perimetry.

	Preoperative evaluation	Operation	3rd day review	3-week review	3-month review
**BCVA**	x		x	x	x
**IOP**	x		x	x	x
**CMT**	x			x	x
**RNFL**	x			x	x
**Flare-cell photometry**	x		x		
**Tolerance**	x		x	x	
**PROM**	x				x

Table 5: Schematic presentation of patient visits. BCVA = best corrected visual acuity, IOP = intraocular pressure, CMT = central macular thickness, RNFL = retinal nerve fiber layer, PROM = patient related outcome measures.

Background literature search

By reviewing the literature, we found no previous studies that compared anti-inflammatory prophylaxis with topical NSAID and prednisolone to topical NSAID alone or to drop-less surgery with perioperative subconjunctival dexamethasone. Neither did the literature answer the question as to whether it is better to start anti-inflammatory prophylaxis preoperatively compared to postoperatively. The literature search revealed a Cochrane review on NSAID

eye drops for treatment of PCME[40] and two systematic reviews and meta-analyses on prevention of PCME after cataract surgery.[2,8] The reviews and meta-analyses did not answer the questions which this study is designed to answer. We also searched www.clinicaltrials.gov for ongoing and unpublished studies but found none similar to ours.

Risks, side effects and inconveniences

The risks of adverse events for patients involved in the study are the same as those for all patients who undergo cataract surgery. According to previous studies, injection of subtenonal steroid is safe and we therefore find that the interventions do not pose any additional risks to the cataract surgery.[22,41] Theoretically though, there is a risk for higher elevations in IOP when a depot of steroid is administered and the participants will be informed about this before deciding whether to participate in the study. The potential consequence of a long lasting elevated IOP is thinning of the RNFL and ultimately visual field defects. Elevated IOP can be lowered using pressure lowering eye drops. Patients in the study will be treated according to the guidelines at our institution if increased pressure is observed. This risk is already a part of the standard treatment and whether it is increased by participation is not currently known. To detect complications and quickly initiate treatment participants are closely monitored throughout the study period by IOP measurements at the postoperative visits and by flare-cell photometry at the 3rd day postoperative visit (Table 5), and all postoperative visits include an evaluation of the eye by slit-lamp imaging. Additional theoretical risks are perforation of the eye and opportunistic infections. Subconjunctival hemorrhage is a common but insignificant side effect and may also occur as a consequence of manipulation of the eye during cataract surgery in itself. The depot may be visible as a small located swelling of the conjunctiva for a limited period of time. To minimize aesthetic inconveniences the depot is placed in the part of the conjunctival sac that is hidden below the lower eyelid. We believe that the risk of adverse effects is counterbalanced by the reduced need for application of eye drops after surgery. The additional examinations will not result in increased risks or complications but may be inconvenient to the patient due to the extra time needed.

Registration of adverse effects

At the postoperative visits patients are checked for adverse effects to the anti-inflammatory prophylaxis. The postoperative visits include measuring of BCVA and IOP and evaluation of the eye by slit-lamp imaging. The patients will also be asked for subjective complaints and symptoms related to the treatment. The number of postoperative visits are illustrated in Table 5. Postoperative visits are scheduled to detect adverse effects when they are expected to present. I.e. the 3rd-day review will detect excessive postoperative inflammation, early elevations in IOP, complications to application of the steroid depot and complications to surgery that were not detected immediately after surgery. The 3-week review will detect elevated IOP and potential infections or corneal defects following prophylactic treatment. The main purpose of the 3-month review is to detect increased CMT which develops within the first 3 months following surgery.[8] Patients will be instructed to contact the investigators immediately if vision gets worse, the eye turns red and/or painful or they become sensitive to light, experience a curtain-like shadow over the visual field, experience flashes of light or halos around light sources. Patients will not be evaluated for adverse effects to

prophylactic treatment beyond the 3-month review but in case the patient experiences an adverse event or reaction within the trial period the patient will be monitored and/or treated for as long as it is relevant given the circumstances. Sponsor-investigator will make the decision.

Adverse effects and reactions

Adverse effect (AE): Any unwanted event regarding a participant in a clinical trial treated with an investigational medicinal product, not necessarily linked to the treatment.

Adverse reaction (AR): Any harmful and unwanted reaction to an investigational medicinal product with no regards to the dose.

Unexpected adverse reaction (UAR): An adverse reaction of which the character or severity does not match the description of adverse reactions in the summary of product characteristics for the given investigational medicinal product.

Serious adverse event (SAE) or serious adverse reaction (SAR): An adverse event or reaction that, regardless of dose, results in death, is life-threatening, causes hospitalization, results in significant or sustained incapacity or leads to a congenital anomaly or malformation.

Suspected unexpected serious adverse reaction (SUSAR): A suspected serious adverse reaction which was not expected.

Reporting adverse effects to the authorities

All serious adverse effects and reactions will be reported to the sponsor-investigator by email. Sponsor-investigator will report any serious adverse event (SAE) unexpected adverse reaction (UAR) and suspected unexpected serious adverse reaction (SUSAR) to the Danish Medicines Agency. SAEs will be reported within 24 hours of recognition to the sponsor-investigator. SUSARs that are mortal or life-threatening will be reported by sponsor-investigator as soon as possible and within 7 days from recognition. All relevant information on follow-up will be reported within 8 days from the initial report. All other SUSARs will be reported within 15 days from recognition. All reports will include a comment on the consequences for the ongoing trial. The attached summary of product characteristics (appendix 1-3) will serve as reference.

Throughout the entire study period a complete list of SARs and SUSARs and a report on the safety of participants will be sent to the Danish Medicines Agency and the National Committee on Health Research Ethics once per year. When the study has completed all results, adverse events and adverse reactions will be entered in the EudraCT within 1 year. The Danish Medicines Agency will be informed within 90 days from termination of the study.

Criteria for aborting the study

The entire study will be aborted if one or more of the interventional groups experience a significantly worse outcome than the other interventional groups regarding development of PCME, IOP elevations ≥ 30 mmHg or unexpected complications.

Interim analyses will be performed when 50 % of the intended study population has completed the 3-month follow-up.

A participant will be withdrawn from the study and treated according to standard procedures at our institution in case of significant complications to surgery such as posterior capsule rupture/vitreous loss, choroidal hemorrhage, dislocated lens material and endophthalmitis. Participants will not be withdrawn from the study due to adverse effects related to prophylactic regimens but prophylactic treatment may be discontinued or accompanied by additional treatment. All adverse effects will be treated according to standard procedures at our institution.

If IOP is elevated above 25 mmHg at any time during the study, pressure lowering treatment is initiated according to standard procedures at our institution which implicates administration of pressure lowering eye drops. If the IOP is not lowered to target level by pressure lowering treatment or if the IOP is elevated beyond 30 mmHg prophylactic treatment with prednisolone eye drops will be discontinued if used. If postoperative inflammation is not sufficiently controlled by the assigned prophylactic regimen at the 3-day follow-up, or if significant ocular inflammation is seen later in the study period the patient is treated according to standard procedures at our institution. Standard procedure is to add steroid eye drops or increase the frequency of steroid eye drops for one week followed by an examination.

Patients who are excluded due to perioperative complications will not provide data for the study. Participants who experience adverse effects during the study will all provide data for the study to determine the rate of complications. Since we did the sample size calculations to account for drop-out, we do not intend to include additional participants to replace excluded participants.

Ethical considerations

The literature contains no answers as to which of the intended treatments is the most effective in preventing PCME and none of the treatments have been described to possess a greater risk of adverse events such as elevated IOP and thinning of the RNFL. Hence we have no preliminary knowledge to indicate if one of the prophylactic regimens would be advantageous to patients. When treating patients with corticosteroids there is a risk for elevated IOP and consequently optic nerve damage. The risk is already a part of the standard regimen and we have no reason to believe that it is increased by participation in the study. In addition we do extensive follow-up on the participants and if IOP increases to more than 25 mmHg pressure lowering treatment is initiated. Studies have shown that NSAID eye drops are better at preventing postoperative inflammation than steroid eye drops[2] and NSAID is part of both eye drop

treatments. The treatment with subtenonal steroid is reportedly safe[22,41] but its' efficacy compared to NSAID alone and combination treatment is not known.

The clinical setup includes time slots for operation assigned to participants in the study. This means that participants in the study may be operated sooner than if they were not participating in the study. There are no other direct benefits related to participation in the study and participation does not change the surgical procedure regarding cataract extraction. The study has no influence on the decision to surgically remove the cataract or not. Participants will contribute to improving the anti-inflammatory prophylaxis of future patients.

The study has the potential to determine which anti-inflammatory prophylactic regimen is most effective and safe after cataract surgery and potentially to reduce the need for eye-drop treatment. If the postoperative anti-inflammatory prophylactic regimen is optimized, the incidence of PCME may fall and more cataract patients may regain perfect vision after surgery. Reducing the need for eye-drop treatment may benefit both patients and society in terms of reduced inconvenience, increased compliance to treatment and reduced costs. We therefore find that the potential advantages outweigh the inconveniences and risks related to participation in the study.

The results of the study will be published as an article in a scientific journal with no regards to whether they are positive, negative or inconclusive.

Time-schedule

The study is scheduled to begin at 1 November 2017 and to finish 1 November 2020. A rough time schedule is presented below in Table 6. The authorities will be informed when the study has finished. If finished at the planned date authorities will be informed within 90 days, if finished earlier they will be informed within 15 days.

June 2017	July 2017	November 2017	February 2018	June 2019	December 2019	April 2020
Application for the Danish Medicines Agency (DMA) (approval granted August 2017)	Application for National Committee on Health Research Ethics (Approval granted November 2017) Application for the Danish Data Protection Agency (Approval granted September 2017)	Preparations	First patient first visit	Last patient last visit	Data analyses completed	Publication of results

Table 6: Time schedule of the study

Quality assurance

The study is monitored by the GCP-Unit at Copenhagen University Hospital, Bispebjerg Hospital, Building 51, 3rd floor, Bispebjerg Bakke 23, 2400 Copenhagen NV. The GCP-Unit and the Danish Health Authority will be allowed direct access to source data during monitoring, auditing and/or inspection. Any information about participants is protected

according to the Danish Act on Processing of Personal Data (Act No. 429 of 31 May 2000) and the Danish Health Act (Act No. 1202 of 14 November 2011). The study is reported to the Danish Medicines Agency, The National Committee on Research Ethics, and the Danish Data Protection Agency. The study will be registered in the EudraCT- and www.clinicaltrials.gov databases and the study will be conducted per protocol and applicable laws and according to ICH-GCP.

Data storage after the study has completed

The data will be stored for 5 years after the study has completed.

Economy

This is an investigator initiated study and we declare no conflicts of interests. We have applied for funding from independent funds and received a total of 2,230,000 DKK. We intend to continue applying for funding through independent funds. The study is performed at the Department of Ophthalmology, Rigshospitalet-Glostrup and is covered by the department's insurance. The study is part of a Ph.D. thesis which has an overall budget of 2,903,750.00 DKK. A budget is presented in Table 7.

**Expenses**			**Grants**	
**Article**	**Amount per year**	**Total amount**	**Funding body**	**Amount**
Salary, PhD student	570000	1710000	Øjenforeningen	350000
Tuition fee	60000	180000	Henry og Astrid Møllers Fond	25000
Materials		85000	Forskningsrådet for Sundhed og Sygdom	1855000
Salary, Optician		430000		
Congresses	20000	60000		
Publications		30000		
Misc. Fees		30000		
Total		2525000		
Overhead, 15%		378750		
Total		2903750		2230000

Table 7: Budget of the Ph.D. thesis. All amounts are in DKK.

References

1. NKR: Behandling af aldersbetinget grå stær. 1–69 (2013). Available at: https://sundhedsstyrelsen.dk/da/udgivelser/2014/nkr-graa-staer. (Accessed: 25th January 2016)

2. Kessel, L. *et al.* Post-cataract prevention of inflammation and macular edema by steroid and nonsteroidal anti-inflammatory eye drops: A systematic review. *Ophthalmology* **121,** 1915–1924 (2014).

3. Kessel, L. Can we meet the future demands for cataract surgery? *Acta Ophthalmol.* **89,** e289-90 (2011).

4. Guo, S. *et al.* Management of pseudophakic cystoid macular edema. *Surv. Ophthalmol.* **60,** 123–137 (2015).

5. Chu, C. J. *et al.* Risk Factors and Incidence of Macular Edema after Cataract Surgery A Database Study of 81984 Eyes. *Ophthalmology* **123,** 316–323 (2016).

6. Belair, M.-L. *et al.* Incidence of Cystoid Macular Edema after Cataract Surgery in Patients with and without Uveitis Using Optical Coherence Tomography. *Am J Ophthalmol* **148,** 128–35 (2009).

7. Kim, S. J., Equi, R. & Bressler, N. M. Analysis of Macular Edema after Cataract Surgery in Patients with Diabetes Using Optical Coherence Tomography. *Ophthalmology* **114,** 881–889 (2007).

8. Wielders, L. H. P. *et al.* Prevention of cystoid macular edema after cataract surgery in nondiabetic and diabetic patients: A systematic review and meta-analysis. *Am. J. Ophthalmol.* **160,** 968–981.e33 (2015).

9. Kim, S. J. *et al.* Topical Nonsteroidal Anti-inflammatory Drugs and Cataract Surgery: A Report by the American Academy of Ophthalmology. *Ophthalmology* **122,** 2159–2168 (2015).

10. An, J. A., Kasner, O., Samek, D. A. & Lévesque, V. Evaluation of eyedrop administration by inexperienced patients after cataract surgery. *J. Cataract Refract. Surg.* **40,** 1857–61 (2014).

11. Winfield, a J., Jessiman, D., Williams, a & Esakowitz, L. A study of the causes of non-compliance by patients prescribed eyedrops. *Br. J. Ophthalmol.* **74,** 477–480 (1990).

12. Semeraro F, Russo A, Gambicorti E, Duse S, Morescalchi F, Vezzoli S, C. C. Efficacy and vitreous levels of topical NSAIDs. *Expert Opin Drug Deliv.* **12,** 1767–82 (2015).

13. Kim, S. J., Flach, A. J. & Jampol, L. M. Nonsteroidal anti-inflammatory drugs in ophthalmology. *Surv. Ophthalmol.* **55,** 108–33 (2010).

14. Schoenberger, S. D., Kim, S. J., Sheng, J. & Calcutt, M. W. Reduction of vitreous prostaglandin E2 levels after topical administration of ketorolac 0.45%. *JAMA Ophthalmol.* **132,** 150–4 (2014).

15. Walters, T., Raizman, M., Ernest, P., Gayton, J. & Lehmann, R. In vivo pharmacokinetics and in vitro pharmacodynamics of nepafenac, amfenac, ketorolac, and bromfenac. *J. Cataract Refract. Surg.* **33,** 1539–

1545 (2007).

16. Bucci, F. A., Waterbury, L. D. & Amico, L. M. Prostaglandin E2 inhibition and aqueous concentration of ketorolac 0.4% (acular LS) and nepafenac 0.1% (nevanac) in patients undergoing phacoemulsification. *Am. J. Ophthalmol.* **144,** 146–7 (2007).

17. Bucci, F. A. & Waterbury, L. D. Prostaglandin E2 inhibition of ketorolac 0.45%, bromfenac 0.09%, and nepafenac 0.1% in patients undergoing phacoemulsification. *Adv. Ther.* **28,** 1089–1095 (2011).

18. Heier, J. S. *et al.* Vitreous nonsteroidal antiinflammatory drug concentrations and prostaglandin E2 levels in vitrectomy patients treated with ketorolac 0.4%, bromfenac 0.09%, and nepafenac 0.1%. *Retina* **29,** 1310–3 (2009).

19. Chang, D. T. W. *et al.* Intracameral dexamethasone reduces inflammation on the first postoperative day after cataract surgery in eyes with and without glaucoma. *Clin. Ophthalmol.* **3,** 345–355 (2009).

20. Gungor, S., Bulam, B., Akman, A. & Colak, M. Comparison of intracameral dexamethasone and intracameral triamcinolone acetonide injection at the end of phacoemulsification surgery. *Indian J. Ophthalmol.* **62,** 861 (2014).

21. Wang, B., Dong, N., Xu, B., Liu, J. & Xiao, L. Efficacy and safety of intracameral triamcinolone acetonide to control postoperative inflammation after phacotrabeculectomy. *J. Cataract Refract. Surg.* **39,** 1691–1697 (2013).

22. Negi, A. K., Browning, A. C. & Vernon, S. A. Single perioperative triamcinolone injection versus standard postoperative steroid drops after uneventful phacoemulsification surgery. Randomized controlled trial. *J. Cataract Refract. Surg.* **32,** 468–474 (2006).

23. Athanasiadis, Y., Tsatsos, M., Sharma, A. & Hossain, P. Subconjunctival triamcinolone acetonide in the management of ocular inflammatory disease. *J Ocul Pharmacol Ther* **29,** 516–522 (2013).

24. Karalezli, A., Borazan, M. & Akova, Y. a. Intracameral triamcinolone acetonide to control postoperative inflammation following cataract surgery with phacoemulsification. *Acta Ophthalmol.* **86,** 183–187 (2008).

25. Karalezli, A., Borazan, M., Kucukerdonmez, C., Akman, A. & Akova, Y. A. Effect of intracameral triamcinolone acetonide on postoperative intraocular pressure after cataract surgery. *Eye (Lond).* **24,** 619–23 (2010).

26. Dieleman, M., Wubbels, J. & Kooten-noordzij, M. Van. Single perioperative subconjunctival steroid depot versus postoperative steroid eyedrops to prevent intraocular inflammation and macular edema after cataract surgery. 1589–1597 (2016). doi:10.1016/j.jcrs.2011.03.049

27. Shorstein, N. H., Liu, L., Waxman, M. D. & Herrinton, L. J. Comparative effectiveness of three prophylactic

strategies to prevent clinical macular edema after phacoemulsification surgery. *Ophthalmology* **122,** 2450–2456 (2015).

28. McGhee, C. N. *et al.* Penetration of synthetic corticosteroids into human aqueous humour. *Eye (Lond).* **4,** 526–30 (1990).

29. Watson, D. G., McGhee, C. N., Midgley, J. M., Dutton, G. N. & Noble, M. J. Penetration of topically applied betamethasone sodium phosphate into human aqueous humour. *Eye (Lond).* **4 (Pt 4),** 603–6 (1990).

30. Weijtens, O. *et al.* Intraocular penetration and systemic absorption after topical application of dexamethasone disodium phosphate. *Ophthalmology* **109,** 1887–1891 (2002).

31. Watson, D., Noble, M. J., Dutton, G. N., Midgley, J. M. & Healey, T. M. Penetration of topically applied dexamethasone alcohol into human aqueous humor. *Arch. Ophthalmol. (Chicago, Ill. 1960)* **106,** 686–7 (1988).

32. Weijtens, O. *et al.* High concentration of dexamethasone in aqueous and vitreous after subconjunctival injection. *Am. J. Ophthalmol.* **128,** 192–197 (1999).

33. Merkoudis, N., Wikberg Matsson, A. & Granstam, E. Comparison of peroperative subconjunctival injection of methylprednisolone and standard postoperative steroid drops after uneventful cataract surgery. *Acta Ophthalmol.* **92,** 623–628 (2014).

34. McGhee, C. N. J., Dean, S. & Danesh-Meyer, H. Locally administered ocular corticosteroids: benefits and risks. *Drug Saf.* **25,** 33–55 (2002).

35. Bartlett, J. D., Woolley, T. W. & Adams, C. M. Identification of high intraocular pressure responders to topical ophthalmic corticosteroids. *J. Ocul. Pharmacol.* **9,** 35–45 (1993).

36. Flach, A. J. Corneal melts associated with topically applied nonsteroidal anti-inflammatory drugs. *Trans. Am. Ophthalmol. Soc.* **99,** 205-10–2 (2001).

37. Kirkwood, B. & Sterne, J. *Essentials : Essential Medical Statistics (2)*. (Wiley-Blackwell, 2003).

38. Sørensen, M. S., Andersen, S., Henningsen, G. O., Larsen, C. T. & Sørensen, T. L. Danish version of Visual Function Questionnaire-25 and its use in age-related macular degeneration. *Dan. Med. Bull.* **58,** A4290 (2011).

39. Lundström, M. *et al.* NIKE: A new clinical tool for establishing levels of indications for cataract surgery. *Acta Ophthalmol. Scand.* **84,** 495–501 (2006).

40. Sivaprasad, S., Bunce, C. & Crosby-Nwaobi, R. Non-steroidal anti-inflammatory agents for treating cystoid macular oedema following cataract surgery. *Cochrane database Syst. Rev.* **2,** CD004239 (2012).

41. Paganelli, F. *et al.* A single intraoperative sub-Tenon's capsule triamcinolone acetonide injection for the

treatment of post-cataract surgery inflammation. *Ophthalmology* **111,** 2102–2108 (2004).
