# Supplementary material for: Prednisolone and Ketorolac vs Ketorolac Monotherapy or Sub-Tenon Prophylaxis for Macular Thickening in Cataract Surgery: A Randomized Clinical Trial
Source: JAMA Ophthalmol. 2021 Aug 12;139(10):1062–70. doi: 10.1001/jamaophthalmol.2021.2976 (PMC8529413; doi:10.1001/jamaophthalmol.2021.2976)
Supplement: Supplement 2. — Statistical Analysis Plan [file jamaophthalmol-e212976-s002.pdf]

Standard Operating Procedure

Plan for statistical analysis

Introduction

This standard operating procedure (SOP) describes the plan for statistical analyses of data collected in the Study for Optimizing Anti-inflammatory Prophylaxis – "SOAP".

The SOP is made in cooperation with associate professor Julie Lyng Forman, Section of Biostatistics, University of Copenhagen, Denmark.

SOAP is a randomized controlled trial consisting of 5 interventional groups (Figure 1). The purpose is to optimize the anti-inflammatory prophylactic treatment administered parallel to cataract surgery.

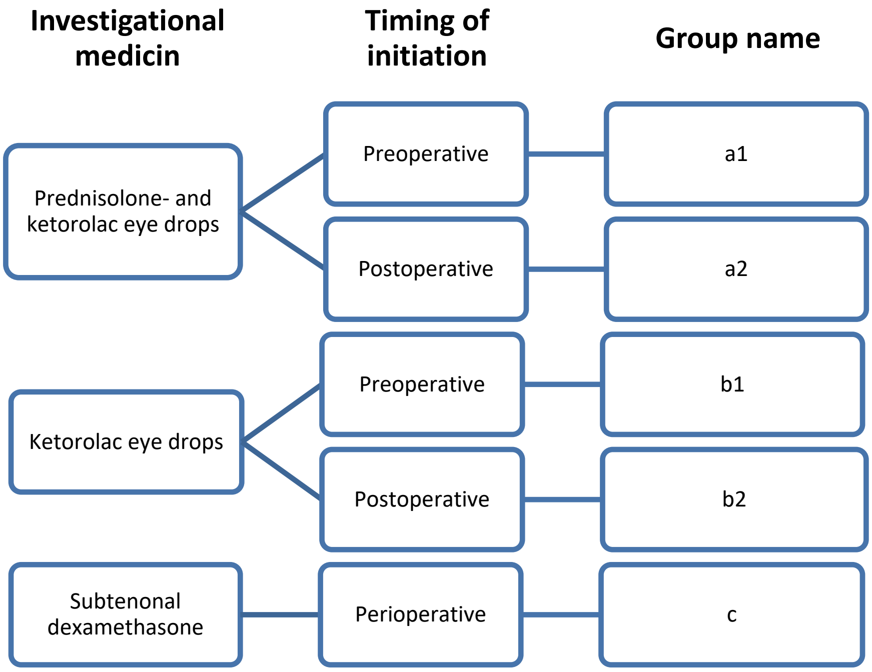

Figure 1: Illustration of the five interventional groups. Preoperative = Initiation three days before surgery; Postoperative = Initiation on the day of surgery; Perioperative = depot placed during surgery.

Group a1 is the control group because it the regimen is similar to the standard regimen at Department of Ophthalmology, Rigshospitalet – Glostrup when the study was designed. A power calculation was made to determine the number of participants in order to be able to detect a 5 micrometer difference on central macular thickness between two groups, since this difference was considered the minimal relevant difference.

The analyses will be performed after renaming the groups by an independent researcher with no attachment to the study. This way the statistical analyses are blinded to allocation. Since we do not know which group is the control group analyses will be repeated with all pseudo groups acting as control group. The correct analyses will be used after unblinding when all analyses are completed. As an exception, secondary analyses in which eye drop groups are combined (explained below) will be done after unblinding.

Primary outcome

The primary outcome is change in central macular thickness from baseline to three-month postoperative visit. We will analyze data using a constrained linear mixed model with unstructured covariance pattern. We will make 4 pairwise comparisons with the control group. To compensate for multiple testing, we will use a Bonferroni correction. This correction makes the level of significance 0.0125 and confidence intervals will be 98.75% confidence intervals.

Secundary outcomes

The secondary outcomes, that are measured repeatedly, will also be analyzed using a constrained linear mixed model with unstructured covariance pattern. These analyses are exploratory and will be corrected with a false detection rate (FDR) method.
As a secondary analyses we will analyze the effects of initiating eye drop preoperatively vs. postoperatively (Groups a1 + b1 vs. groups a2 + b2) and combination treatment vs. ketorolac alone (Groups a1 + a2 vs. b1 + b2). These analyses will be performed on primary as well as secondary outcomes.

Regarding publication plan: We intend to publish a main article with primary outcome and data from three-week- and three-month visits ("late postoperative effects of treatment") and an article with data from the three-days visit ("early postoperative effects of treatment"). In the latter the primary analysis will be change in anterior chamber flare.

Adverse events

Adverse events (AE) will be counted and presented as numbers and percentages. We will make pairwise comparisons with the control group by using Fishers' exact test. Evaluation will primarily be qualitative and we will use an FDR-method to adjust for multiple comparisons.

Missing data

The primary analysis will be done according to the principle "intention to treat" (ITT). Hence, all participants who contribute with baseline data will provide data for the analyses. Only participants who were excluded do not provide data for the analyses. Mixed model imputates missing data under the assumption of "missing at random".

As supplements to the ITT-analyses, the following sensitivity-analyses will be performed:

1. Best case/worst case scenarios using 10% and 90% quantiles
2. "per protocol"

Finally, we will investigate if dropout is associated with adverse events, adverse reactions or patient characteristics using descriptive statistics.

Restrictions for timing of visits

We define certain intervals for timing of postoperative visits to ensure that it is reasonable to compare three-days-, three-week- and three-month visits, respectively.

- A three-days visit must be 2-4 days from surgery, including both days
- A three-week visit must be 14-28 days from surgery, including both days
- A three-month visit must be 60-120 days from surgery, including both days

Data is not included in the analysis if these time-limits are not met.

Management of extreme outliers

To determine the significance of extreme outliers, analyses will be performed both with and without these.
