# Supplementary material for: Prednisolone and Ketorolac vs Ketorolac Monotherapy or Sub-Tenon Prophylaxis for Macular Thickening in Cataract Surgery: A Randomized Clinical Trial
Source: JAMA Ophthalmol. 2021 Aug 12;139(10):1062–70. doi: 10.1001/jamaophthalmol.2021.2976 (PMC8529413; doi:10.1001/jamaophthalmol.2021.2976)
Supplement: Supplement 3. — eFigure. Intraocular Pressure From Baseline to 3 Months After Surgery eTable 1. Best Case Control Analyses eTable 2. Worst Case Control Analyses eTable 3. Per-Protocol Analyses eTable 4. Analyses Without Extreme Outliers eTable 5. Analyses With Truncated Extreme Outliers eTable 6. Analyses for Postrandomization Confounding eTable 7. Reasons for Exclusion eTable 8. Number of Participants With CST Increase ≥10% eTable 9. Combination vs NSAID Monotherapy eTable 10. Preoperative vs Postoperative Initiation of Prophylactic Treatment eTable 11. Baseline Characteristics for Completers, Noncompleters, and Drop-outs [file jamaophthalmol-e212976-s003.pdf]

Supplementary Online Content

Erichsen JH, Holm LM, Forslund Jacobsen M, Forman JL, Kessel L. Prednisolone and ketorolac vs ketorolac monotherapy or sub-Tenon prophylaxis for macular thickening in cataract surgery: a randomized clinical trial. Published online August 12, 2021. *JAMA Ophthlamol*. doi:10.1001/jamaophthalmol.2021.2976

eFigure. Intraocular Pressure From Baseline to 3 Months After Surgery

eTable 1. Best Case Control Analyses

eTable 2. Worst Case Control Analyses

eTable 3. Per-Protocol Analyses

eTable 4. Analyses Without Extreme Outliers

eTable 5. Analyses With Truncated Extreme Outliers

eTable 6. Analyses for Postrandomization Confounding

eTable 7. Reasons for Exclusion

eTable 8. Number of Participants With CST Increase ≥10%

eTable 9. Combination vs NSAID Monotherapy

eTable 10. Preoperative vs Postoperative Initiation of Prophylactic Treatment

eTable 11. Baseline Characteristics for Completers, Noncompleters, and Drop-outs

This supplementary material has been provided by the authors to give readers additional information about their work.

© 2021 Erichsen JH, et al. *JAMA Ophthalmology*.

eFigure. Intraocular Pressure From Baseline to 3 Months After Surgery

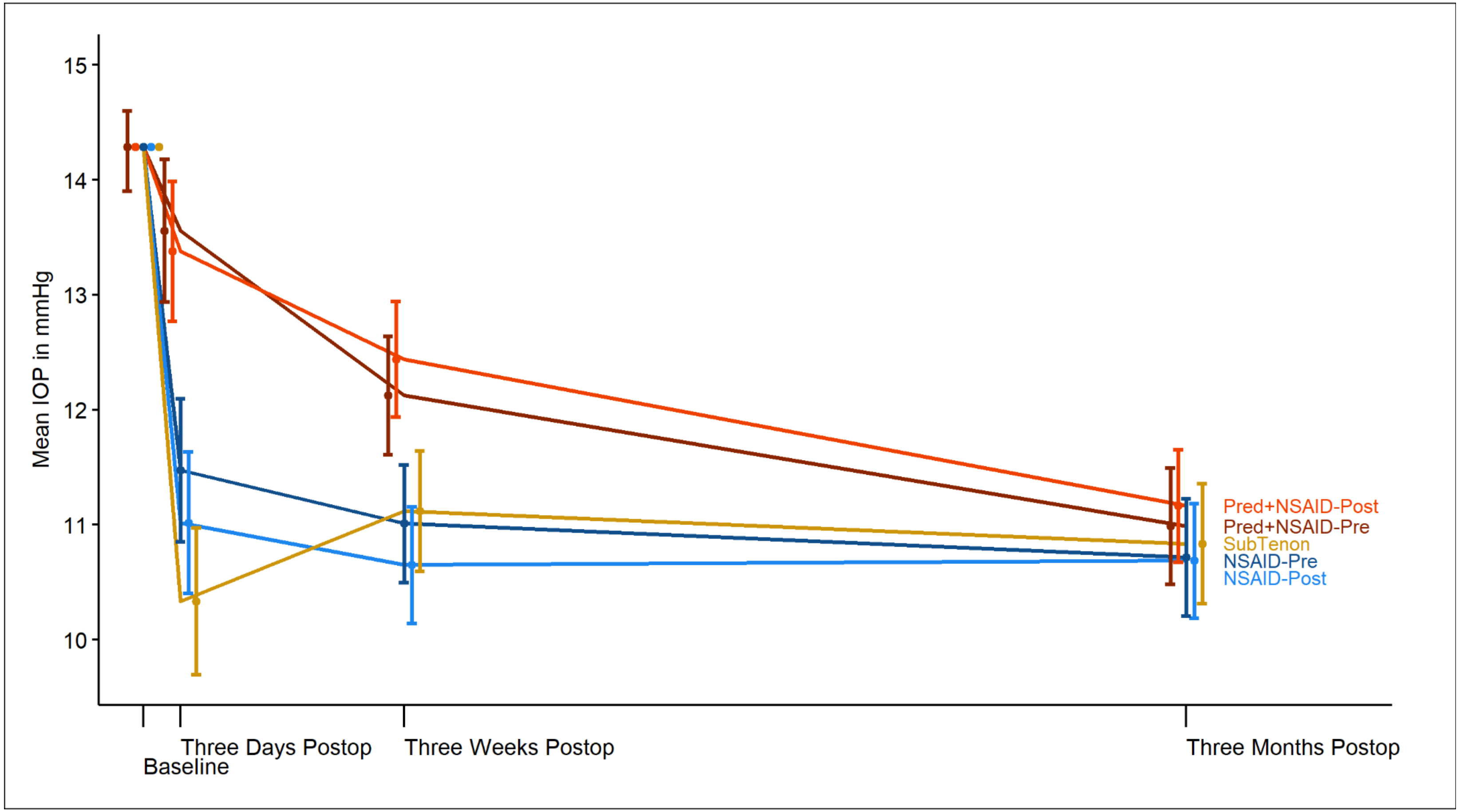

Error bars represent 95% confidence intervals. Only one error bar is presented at baseline since estimates per definition are the same for all groups in the constrained linear mixed model with inherent baseline adjustment. IOP = intraocular pressure; Postop = postoperative.

© 2021 Erichsen JH, et al. *JAMA Ophthalmology*.

eTable 1. Best Case Control Analyses

	Pred+NSAID-Pre	Pred+NSAID-Post	NSAID-Pre	NSAID-Post	SubTenon
**CST, microns**					
Baseline	243.2 [241.2; 245.2]	-	-	-	-
Three weeks postop	+3.9 [+0.2; +7.6]	+4.8 [-0.4; +10.0]	+5.8 [0.5; +11.1]	+3.4 [-1.8; +8.7]	+14.2 [+8.9; +19.5]
p		.07	.03	.20	<.001
Three months postop	+5.5 [+2.0; +9.0]	+2.3 [-2.7; +7.2]	+6.1 [+1.1; +11.1]	+2.9 [-2.1; +7.9]	+9.5 [+4.5; +14.5]
p		.37	.02	.25	<.001
**IOP, mm Hg**					
Baseline	14.3 [13.9; 14.6]	-	-	-	-
Three days postop	-0.9 [-1.5; -0.2]	+0.2 [-0.7; +1.1]	-1.7 [-2.6; -0.9]	-2.1 [-3.0; -1.3]	-2.6 [-3.5; -1.7]
p		.68	<.001	<.001	<.001
Three weeks postop	-2.3 [-2.9; -1.8]	+0.5 [-0.1; +1.2]	-0.9 [-1.6; -0.2]	-1.2 [-1.9; -0.5]	-0.6 [-1.3; +0.1]
p		.12	.01	<.001	.08
Three months postop	-3.5 [-4.0; -2.9]	+0.4 [-0.2; +1.1]	+0.3 [-0.4; +1.0]	+0.0 [-0.6; +0.7]	+0.4 [-0.3; +1.1]
p		.20	.38	.89	.23
**CDVA, logMAR**					
Baseline	0.29 [0.28; 0.30]	-	-	-	-
Three days postop	-0.19 [-0.30; -0.16]	+0.01 [-0.04; +0.05]	-0.02 [-0.07; +0.02]	+0.01 [-0.04; +0.05]	+0.03 [-0.02; +0.07]
p		.75	.32	.75	.26
Three weeks postop	-0.28 [-0.31; -0.25]	+0.02 [-0.01; +0.05]	+0.01 [-0.02; +0.04]	+0.01 [-0.03; +0.04]	+0.05 [+0.01; +0.08]
p		.21	.58	.72	.006
Three months postop	-0.31 [-0.34; -0.29	+0.02 [-0.01; +0.05]	+0.03 [+0.00; +0.06]	+0.01 [-0.01; +0.04]	+0.04 [+0.01; +0.06]
p		.11	.04	.33	.01

In the "best case control" analyses, missing values were substituted with the 10th percentile of the observed data in the control group and the 90th percentile in the rest of the groups. All estimates were derived from the constrained linear mixed model with inherent baseline adjustment. Values for Pred+NSAID-Pre (control group) are presented as change from baseline and values for Pred+NSAID-Post, NSAID-Pre, NSAID-Post and SubTenon are presented as differences from Pred+NSAID-Pre (estimated treatment difference). The baseline value was the same for all groups. All estimates are presented with 95% confidence intervals. Postop = postoperative; CST = central subfield thickness; IOP = intraocular pressure; CDVA = corrected distance visual acuity; logMAR = logarithm to the minimal angle of resolution. P-values were not corrected for multiple testing.

© 2021 Erichsen JH, et al. *JAMA Ophthalmology*.

eTable 2. Worst Case Control Analyses

	Pred+NSAID-Pre	Pred+NSAID-Post	NSAID-Pre	NSAID-Post	SubTenon
**CST, microns**					
Baseline	243.2 [241.2; 245.2]	-	-	-	-
Three weeks postop	+7.4 [+3.5; +11.3]	-0.7 [-6.2; +4.7]	+0.9 [-4.6; +6.5]	-1.4 [-6.9; +4.1]	+6.4 [+0.9; +12.0]
p		.79	.74	.62	.02
Three months postop	+9.5 [+6.2; +12.8]	-2.8 [-7.4; +1.8]	-2.7 [-7.4; +2.0]	-4.5 [-9.1; +0.2]	-0.1 [-4.8; +4.6]
p		.24	.26	.06	.96
**IOP, mm Hg**					
Baseline	14.3 [13.9; 14.6]	-	-	-	-
Three days postop	-0.6 [-1.2; +0.0]	-0.4 [-1.3; +0.4]	-2.4 [-3.2; -1.5]	-2.8 [-3.6; -1.9]	-3.6 [-4.4; -2.7]
p		.33	<.001	<.001	<.001
Three weeks postop	-2.0 [-2.5; -1.4]	-0.1 [-0.8; +0.6]	-1.4 [-2.1; -0.7]	-1.7 [-2.4; -1.0]	-1.4 [-2.2; -0.7]
p		.87	<.001	<.001	<.001
Three months postop	-3.0 [-3.5; -2.5]	-0.2 [-0.8; +0.5]	-0.8 [-1.4; -0.1]	-0.7 [-1.4; -0.1]	-0.8 [-1.5; -0.2]
p		.61	.02	.03	.01
**CDVA, logMAR**					
Baseline	0.29 [0.28; 0.30]	-	-	-	-
Three days postop	-0.18 [-0.22; -0.15]	-0.02 [-0.06; +0.03]	-0.05 [-0.09; +0.00]	-0.01 [-0.06; +0.03]	-0.01 [-0.05; +0.04]
p		.41	.046	.51	.72
Three weeks postop	-0.26 [-0.29; -0.24]	+0.00 [-0.04; +0.03]	-0.01 [-0.04; +0.02]	-0.02 [-0.05; +0.02]	+0.02 [-0.02; +0.05]
p		.88	.48	.36	.33
Three months postop	-0.29 [-0.32; -0.27]	-0.01 [-0.03; +0.02]	-0.01 [-0.04; +0.02]	-0.01 [-0.04; +0.01]	+0.00 [-0.03; +0.03]
p		.65	.45	.32	.90

In the "worst case control" analyses, missing values were substituted with the 90th percentile of the observed data in the control group and the 10th percentile in the rest of the groups. All estimates were derived from the constrained linear mixed model with inherent baseline adjustment. Values for Pred+NSAID-Pre (control group) are presented as change from baseline and values for Pred+NSAID-Post, NSAID-Pre, NSAID-Post and SubTenon are presented as differences from Pred+NSAID-Pre (estimated treatment difference). The baseline value was the same for all groups. All estimates are presented with 95% confidence intervals. Postop = postoperative; CST = central subfield thickness; IOP = intraocular pressure; CDVA = corrected distance visual acuity; logMAR = logarithm to the minimal angle of resolution. P-values were not corrected for multiple testing.

© 2021 Erichsen JH, et al. *JAMA Ophthalmology*.

eTable 3. Per-Protocol Analyses

	Pred+NSAID-Pre	Pred+NSAID-Post	NSAID-Pre	NSAID-Post	SubTenon
**CST, microns**					
Baseline	242.1 [239.9; 244.3]	-	-	-	-
Three weeks postop	+5.0 [+3.8; +6.3]	-0.9 [-2.6; +0.9]	+2.3 [+0.5; +4.1]	+1.5 [-0.2; +3.2]	+5.7 [+3.4; +7.9]
p		.32	.01	.09	<.001
Three months postop	+7.1 [+5.8; +8.4]	-1.7 [-3.6; +0.1]	-0.2 [-2.2; +1.7]	-1.3 [-3.2; +0.5]	+0.6 [-1.7; +3.0]
p		.07	.81	.16	.59
**IOP, mm Hg**					
Baseline	14.5 [14.1; 14.8]	-	-	-	-
Three days postop	-0.7 [-1.4; -0.1]	-0.1 [-1.1; +0.8]	-2.3 [-3.2; -1.3]	-2.6 [-3.5; -1.7]	-2.6 [-3.8; -1.4]
p		.79	<.001	<.001	<.001
Three weeks postop	-2.2 [-2.8; -1.7]	+0.5 [-0.2; +1.3]	-1.2 [-1.9; -0.4]	-1.4 [-2.1; -0.7]	-1.0 [-1.9; -0.1]
p		.15	.002	<.001	.04
Three months postop	-3.4 [-3.9; -2.9]	+0.3 [-0.4; +0.9]	-0.5 [-1.2; +0.2]	-0.5 [-1.2; +0.1]	-0.2 [-1.0; +0.7]
p		.44	.18	.11	.71
**CDVA, logMAR**					
Baseline	0.29 [0.28; 0.31]	-	-	-	-
Three days postop	-0.21 [-0.25; -0.18]	+0.00 [-0.04; +0.04]	-0.02 [-0.07; +0.02]	+0.02 [-0.02; +0.06]	+0.00 [-0.06; +0.05]
p		.95	.26	.32	.93
Three weeks postop	-0.28 [-0.31; -0.26]	+0.01 [-0.02; +0.04]	+0.01 [-0.03; +0.04]	+0.01 [-0.02; +0.04]	+0.02 [-0.02; +0.06]
p		.66	.71	.67	.33
Three months postop	-0.31 [-0.33; -0.28]	+0.00 [-0.03; +0.03]	+0.01 [-0.02; +0.04]	+0.00 [-0.02; +0.03]	+0.02 [-0.02; +0.05]
p		.93	.52	.79	.39

These analyses included only participants who followed the treatment protocol. Thus, participants who received additional anti-inflammatory treatment or reported significant deviations from allocated prophylactic treatment (judged by investigator) were excluded from these analyses. All estimates were derived from the constrained linear mixed model with inherent baseline adjustment. Values for Pred+NSAID-Pre (control group) are presented as change from baseline and values for Pred+NSAID-Post, NSAID-Pre, NSAID-Post and SubTenon are presented as differences from Pred+NSAID-Pre (estimated treatment difference). The baseline value was the same for all groups. All estimates are presented with 95% confidence intervals. Postop = postoperative; CST = central subfield thickness; IOP = intraocular pressure; CDVA = corrected distance visual acuity; logMAR = logarithm to the minimal angle of resolution. P-values were not corrected for multiple testing.

© 2021 Erichsen JH, et al. *JAMA Ophthalmology*.

eTable 4. Analyses Without Extreme Outliers

	Pred+NSAID-Pre	Pred+NSAID-Post	NSAID-Pre	NSAID-Post	SubTenon
**CST, microns**					
Baseline	243.2 [241.2; 245.2]	-	-	-	-
Three weeks postop	+5.3 [+3.6; +7.0]	-0.8 [-3.2; +1.5]	+2.8 [0.5; 5.2]	+1.3 [-1.0; 3.7]	+9.6 [7.2; +12.0]
p		.49	.02	.27	<.001
Three months postop	+7.5 [+5.9; +9.0]	-1.2 [-3.4; +1.0]	+0.6 [-1.6; 2.8]	-1.4 [-3.6; 0.7]	+1.7 [-0.5; +3.9]
p		.28	.58	.20	.14
**CDVA, logMAR**					
Baseline	0.29 [0.27; 0.30]	-	-	-	-
Three days postop	-0.19 [-0.23; -0.16]	+0.00 [-0.04; +0.04]	-0.02 [-0.07; +0.02]	+0.00 [-0.04; +0.05]	+0.02 [-0.03; +0.06]
p		.93	.26	.83	.45
Three weeks postop	-0.26 [-0.29; -0.24]	+0.01 [-0.03; +0.04]	+0.00 [-0.04; +0.03]	-0.01 [-0.04; +0.03]	+0.03 [-0.00; +0.06]
p		.66	.91	.74	.08
Three months postop	-0.30 [-0.32; -0.27]	+0.01 [-0.02; +0.04]	+0.01 [-0.02; +0.04]	+0.00 [-0.03; +0.03]	+0.02 [-0.01; +0.05]
p		.60	.49	>.99	.20

In the analyses without extreme outliers, central subfield thickness > 350 microns (6 extreme outliers) and corrected distance visual acuity > 0.80 logMAR (4 extreme outliers) were removed from the analyses. All estimates were derived from the constrained linear mixed model with inherent baseline adjustment. Values for Pred+NSAID-Pre (control group) are presented as change from baseline and values for Pred+NSAID-Post, NSAID-Pre, NSAID-Post and SubTenon are presented as differences from Pred+NSAID-Pre (estimated treatment difference). The baseline value was the same for all groups. All estimates are presented with 95% confidence intervals. Postop = postoperative; CST = central subfield thickness; IOP = intraocular pressure; CDVA = corrected distance visual acuity; logMAR = logarithm to the minimal angle of resolution. P-values were not corrected for multiple testing.

© 2021 Erichsen JH, et al. *JAMA Ophthalmology*.

eTable 5. Analyses With Truncated Extreme Outliers

	Pred+NSAID-Pre	Pred+NSAID-Post	NSAID-Pre	NSAID-Post	SubTenon
**CST, microns**					
Baseline	243.2 [241.2; 245.2]	-	-	-	-
Three weeks postop	+5.3 [+3.6; +7.0]	-1.0 [-3.4; +1.4]	+2.9 [+0.5; +5.3]	+1.3 [-1.0; +3.7]	+9.6 [+7.2; 12.0]
p		.41	.02	.27	<.001
Three months postop	+7.5 [+5.8; +9.1]	-1.4 [-3.7; +1.0]	+0.6 [-1.8; +3.0]	-1.4 [-3.8 ; +0.9]	+2.4 [+0.0; +4.8]
p		.25	.60	.24	.046
**CDVA, logMAR**					
Baseline	0.29 [0.27; 0.30]	-	-	-	-
Three days postop	-0.19 [-0.22; -0.16]	+0.00 [-0.04; +0.04]	-0.03 [-0.07; +0.02]	+0.00 [-0.04; +0.04]	+0.01 [-0.03; +0.06]
p		.96	.20	.93	.53
Three weeks postop	-0.27 [-0.29; -0.24]	+0.01 [-0.03; +0.04]	+0.00 [-0.03; +0.03]	-0.01 [-0.04; +0.03]	+0.03 [+0.00; +0.06]
p		.64	.92	.76	.08
Three months postop	-0.30 [-0.32; -0.28]	+0.01 [-0.02; +0.04]	+0.01 [-0.02; +0.04]	+0.00 [-0.03; +0.03]	+0.02 [-0.01; +0.05]
p		.58	.49	.99	.19

In the analyses with truncated extreme outliers, central subfield thickness > 350 microns (6 extreme outliers) and corrected distance visual acuity > 0.80 logMAR (4 extreme outliers) were truncated to the mean value of all observed values + 2 standard deviations. All estimates were derived from the constrained linear mixed model with inherent baseline adjustment. Values for Pred+NSAID-Pre (control group) are presented as change from baseline and values for Pred+NSAID-Post, NSAID-Pre, NSAID-Post and SubTenon are presented as differences from Pred+NSAID-Pre (estimated treatment difference). The baseline value was the same for all groups. All estimates are presented with 95% confidence intervals. Postop = postoperative; CST = central subfield thickness; IOP = intraocular pressure; CDVA = corrected distance visual acuity; logMAR = logarithm to the minimal angle of resolution. P-values were not corrected for multiple testing.

© 2021 Erichsen JH, et al. *JAMA Ophthalmology*.

eTable 6. Analyses for Postrandomization Confounding

	Baseline	Three weeks postop	Three months postop
**Central subfield thickness (Microns)**			
Effect of allocation to Pred+NSAID-Pre	N/A	+7.1 [-4.8; +19.0]	-0.3 [-14.9; +14.3]
p		.24	.97
Effect of allocation to Pred+NSAID-Post	N/A	+6.3 [-5.5; +18.1]	-1.7 [-16.2; +12.8]
p		.30	.82
Effect of allocation to NSAID-Pre	N/A	+10.5 [-1.1; +22.1]	+0.4 [-13.8; +14.7]
p		.08	.95
Effect of allocation to NSAID-Post	N/A	+8.2 [-3.6; +20.0]	-1.7 [-16.2; +12.8]
p		.17	.82
Effect of allocation to SubTenon	N/A	+18.6 [+7.0; +30.3]	+4.3 [-10.1; +18.6]
p		.002	.56
Effect of age per one-year increase	-0.1 [-0.4; +0.1]	+0.0 [-0.2; +0.2]	+0.1 [-0.1; +0.3]
p	.32	.98	.20
Effect of being male	+13.3 [+9.3; +17.3]	-0.1 [-2.3; +2.1]	+1.1 [-1.7; +3.8]
p	<.001	.92	.45
Effect of doubling CDE	-1.7 [-4.2; +0.8]	-0.6 [-2.0; +0.8]	-0.7 [-2.4; +1.1]
p	.18	.41	.46

This eTable presents results from the constrained linear mixed model with inherent baseline adjustment when age, sex and CDE were included as covariates. These covariates were included to test if they were possible confounders of the effect of allocation to anti-inflammatory regimen on postoperative central subfield thickness (CST). Estimated effects on CST at baseline, three weeks postoperative and three months postoperative are presented as mean [CI]. CDE = cumulative dissipated energy ("phaco energy"); N/A = no estimate available; CI = 95% confidence interval. P-values were not corrected for multiple testing.

© 2021 Erichsen JH, et al. *JAMA Ophthalmology*.

eTable 7. Reasons for Exclusion

Subject	Exclusion category	Reason for exclusion	Time of exclusion	Allocated regimen initiated?
1	Surgical complication	Residual lens material	First postoperative visit	yes
2	Surgical complication	Residual lens material	First postoperative visit	yes
3	Surgical complication	Residual lens material	First postoperative visit	yes
4	Surgical complication	Residual lens material	First postoperative visit	yes
5	Surgical complication	Toric IOL needed repositioning	First postoperative visit	yes
6	Surgical complication	Toric IOL needed repositioning	First postoperative visit	yes
7	Surgical complication	Posterior capsule rupture	Day of surgery	yes
8	Surgical complication	Posterior capsule rupture	Day of surgery	yes
9	Surgical complication	Retinal detachment	Second postoperative visit	yes
10	Other	Surgeon with < 1000 surgeries performed	Day of surgery	yes
11	Other	Surgeon with < 1000 surgeries performed	Day of surgery	yes
12	Other	Retinal condition recognized after allocation	Day of allocation	no
13	Other	Retinal condition recognized after allocation	Second postoperative visit	yes
14	Other	Indication for surgery on study eye lost after surgery on first eye	Before surgery	no

Residual lens material was found in 4 participants, 2 participants needed repositioning of their toric IOL, 2 participants had a ruptured posterior capsule, 1 participant had a retinal detachment which was found at the second postoperative visit (three weeks postoperatively) and was vitrectomized, 2 participants were operated by a surgeon with < 1000 cataract surgeries performed within the past 2 years due to administrational errors, 1 participant (subject 12) had an epiretinal membrane with vitreomacular traction which was recognized after allocation, 1 participant (subject 13) had exudative age-related macular degeneration which was not recognized at the preoperative visit due to dense cataract, and 1 participant had no subjective symptoms of cataract on the study eye (second eye) after surgery on the first eye.

© 2021 Erichsen JH, et al. *JAMA Ophthalmology*.

eTable 8. Number of Participants With CST Increase ≥ 10%

	Pred+NSAID-Pre	Pred+NSAID-Post	NSAID-Pre	NSAID-Post	SubTenon
**Three weeks, N (%)**	0 (0)	1 (1.1)	4 (4.4)	0 (0)	16 (18.0)
p		>.99	.06	>.99	<.001
**Three months, N (%)**	1 (1.1)	3 (3.2)	6 (6.7)	0 (0)	3 (3.4)
p		.62	.06	>.99	.36

Post-hoc analyses of number of participants with an increased central subfield thickness of at least 10% from baseline. Pairwise comparisons with the control group (Pred+NSAID-Pre) were made with Fisher's exact test. P-values were not corrected for multiple testing.

© 2021 Erichsen JH, et al. *JAMA Ophthalmology*.

eTable 9. Combination vs NSAID Monotherapy

	Pred+NSAID	NSAID	SubTenon
**CST, microns**			
Baseline	243.2 [241.2; 245.2]	-	-
Three weeks postop	+6.8 [+4.2; +9.4]	+0.9 [-2.6; +4.5]	+9.7 [+5.2; +14.1]
p / adj-p		.61 / .89	<.001 / <.001
Three months postop	+7.5 [+5.4; +9.6]	-0.5 [-3.5; +2.6]	+4.5 [+0.7; +8.3]
p / adj-p		.76 / .98	.02 / .05
**IOP, mm Hg**			
Baseline	14.3 [13.9; 14.6]	-	-
Three days postop	-0.8 [-1.3; -0.4]	-2.2 [-2.8; -1.6]	-3.1 [-3.9; -2.4]
p / adj-p		<.001 / <.001	<.001 / <.001
Three weeks postop	-2.0 [-2.4; -1.6]	-1.5 [-1.9; -1.0]	-1.2 [-1.8; -0.6]
p / adj-p		<.001 / <.001	<.001 / <.001
Three months postop	-3.2 [-3.6; -2.8]	-0.4 [-0.8; +0.1]	-0.2 [-0.8; +0.3]
p / adj-p		.10 / .24	.40 / .69
**CDVA, logMAR**			
Baseline	0.29 [0.28; 0.30]	-	-
Three days postop	-0.19 [-0.22; -0.17]	-0.02 [-0.05; +0.02]	+0.01 [-0.03; +0.05]
p / adj-p		.32 / .61	.57 / .85
Three weeks postop	-0.27 [-0.29; -0.25]	-0.01 [-0.03; +0.02]	+0.03 [+0.00; +0.06]
p / adj-p		.51 / .79	.07 / .17
Three months postop	-0.30 [-0.32; -0.28]	+0.00 [-0.02; +0.02]	+0.02 [-0.01; +0.04]
p / adj-p		.95 / >.99	.23 / .47

All estimates were derived from the constrained linear mixed model with inherent baseline adjustment. Values for Pred+NSAID-Pre (control group) are presented as change from baseline and values for Pred+NSAID-Post, NSAID-Pre, NSAID-Post and SubTenon are presented as differences from Pred+NSAID-Pre (estimated treatment difference). The baseline value was the same for all groups. All estimates are presented with 95% confidence intervals. Postop = postoperative; CST = central subfield thickness; IOP = intraocular pressure; CDVA = corrected distance visual acuity; logMAR = logarithm to the minimal angle of resolution; adj-p-value = p-value after adjusting for false discovery rate (FDR). Pred+NSAID was formed by pooling Pred+NSAID-Pre and Pred+NSAID-Post. NSAID was formed by pooling NSAID-Pre and NSAID-Post.

© 2021 Erichsen JH, et al. *JAMA Ophthalmology*.

eTable 10. Preoperative vs Postoperative Initiation of Prophylactic Treatment

	Preoperative	Postoperative	SubTenon
**CST, microns**			
Baseline	243.2 [241.2; 245.2]	-	-
Three weeks postop	+7.2 [+4.6; +9.8]	+0.2 [-3.4; +3.8]	+9.3 [+4.8; +13.8]
p / adj-p		.91 / >.99	<.001 / <.001
Three months postop	+7.8 [+5.6; +10.0]	-1.0 [-4.0; +2.1]	+4.2 [+0.4; +8.1]
p / adj-p		.53 / .81	.03 / .08
**IOP, mm Hg**			
Baseline	14.3 [13.9; 14.6]	-	-
Three days postop	-1.8 [-2.2; -1.3]	-0.3 [-0.9; +0.3]	-2.2 [-3.0; -1.4]
p / adj-p		.34 / .64	<.001 / <.001
Three weeks postop	-2.7 [-3.1; -2.3]	+0.0 [-0.5; +0.5]	-0.4 [-1.1; +0.2]
p / adj-p		.97 / >.99	.16 / .35
Three months postop	-3.4 [-3.8; -3.0]	+0.1 [-0.4; +0.5]	0.0 [-0.6; +0.6]
p / adj-p		.74 / .96	.96 / >.99
**CDVA, logMAR**			
Baseline	0.29 [0.28; 0.30]	-	-
Three days postop	-0.21 [-0.23; -0.18]	+0.01 [-0.02; +0.04]	+0.03 [-0.01; +0.07]
p / adj-p		.45 / .74	.21 / .44
Three weeks postop	-0.27 [-0.29; -0.25]	+0.00 [-0.02; +0.03]	+0.03 [+0.00; +0.06]
p / adj-p		.78 / >.99	.03 / .08
Three months postop	-0.30 [-0.32; -0.28]	+0.00 [-0.02; +0.02]	+0.01 [-0.01; +0.04]
p / adj-p		.98 / >.99	.24 / .49

All estimates were derived from the constrained linear mixed model with inherent baseline adjustment. Values for Pred+NSAID-Pre (control group) are presented as change from baseline and values for Pred+NSAID-Post, NSAID-Pre, NSAID-Post and SubTenon are presented as differences from Pred+NSAID-Pre (estimated treatment difference). The baseline value was the same for all groups. All estimates are presented with 95% confidence intervals. Postop = postoperative; CST = central subfield thickness; IOP = intraocular pressure; CDVA = corrected distance visual acuity; logMAR = logarithm to the minimal angle of resolution; adj-p-value = p-value after adjusting for false discovery rate (FDR). The group “Preoperative” was formed by pooling the groups Pred+NSAID-Pre and NSAID-Pre. The group “Postoperative was formed by pooling the groups Pred+NSAID-Post and NSAID-Post.

© 2021 Erichsen JH, et al. *JAMA Ophthalmology*.

eTable 11. Baseline Characteristics for Completers, Noncompleters, and Drop-outs

	**All**	**Completers**	**Non-completers**	**Drop-outs**
N	470	434	36	22
Sex, female (%)	290 (62)	272 (63)	18 (50)	11 (50)
Sex, male (%)	180 (38)	162 (37)	18 (50)	11 (50)
Age, mean (SD)	72.2 (7.0)	71.9 (6.9)	75.1 (8.1)	76.7 (8.0)
CST, mean (SD)	243.2 (21.8)	243.4 (21.8)	240.0 (21.0)	239.0 (20.0)
CDVA, mean (SD)	0.29 (0.15)	0.29 (0.16)	0.29 (0.12)	0.28 (0.11)
IOP, mean (SD)	14.3 (3.9)	14.3 (3.9)	14.2 (3.3)	14.5 (3.4)
AREDS, median (range)	2.0 (<1.0; >3.0)	2.0 (<1.0; >3.0)	2.5 (1.5; 3.0)	2.0 (1.5; 3.0)

Completers = participants who completed the study; Non-completers = All participants who did not complete the study including those excluded after allocation and those who dropped out; Drop-outs = participants who withdrew before surgery or were lost to follow-up. N = number of participants; CST = central subfield thickness; CDVA = corrected distance visual acuity in logMAR, IOP = intraocular pressure in mm Hg; AREDS = Age-Related Eye Disease Study classification of cataract score for nuclear cataract.

© 2021 Erichsen JH, et al. *JAMA Ophthalmology*.
