# Supplementary material for: Prednisolone and Ketorolac vs Ketorolac Monotherapy or Sub-Tenon Prophylaxis for Macular Thickening in Cataract Surgery: A Randomized Clinical Trial
Source: JAMA Ophthalmol. 2021 Aug 12;139(10):1062–70. doi: 10.1001/jamaophthalmol.2021.2976 (PMC8529413; doi:10.1001/jamaophthalmol.2021.2976)
Supplement: Supplement 4. — Data Sharing Statement [file jamaophthalmol-e212976-s004.pdf]

Data Sharing Statement

Erichsen. Prednisolone and Ketorolac vs Ketorolac Monotherapy or Sub-Tenon Prophylaxis for Macular Thickening in Cataract Surgery. *JAMA Ophthalmol*. Published August 12, 2021. doi:10.1001/jamaophthalmol.2021.2976

Data
Data available: Yes
Data types: Deidentified participant data, Data dictionary
How to access data: jesper.h.erichsen@dadlnet.dk
When available: beginning date: 11-01-2027

Supporting Documents
Document types: Statistical/analytic code
How to access documents: jesper.h.erichsen@dadlnet.dk
When available: beginning date: 11-01-2027

Additional Information
Who can access the data: Data will be made available upon reasonable request.
Types of analyses: Data will be made available for reproduction of our results. Other purposes will be individually evaluatet.
Mechanisms of data availability: After approval of a proposal
Any additional restrictions: Data can only be used in anonymized form and will be made available upon reasonable request after anonymization.
